# Measuring the perception and metacognition of time

**DOI:** 10.1167/jov.24.3.5

**Published:** 2024-03-20

**Authors:** Simon J. Cropper, Daniel R. Little, Liheng Xu, Aurelio M. Bruno, Alan Johnston

**Affiliations:** 1 Melbourne School of Psychological Sciences, University of Melbourne, Melbourne, Australia; 2 Department of Psychology, University of York, York, UK; 3 School of Psychology and Vision Sciences, University of Leicester, Leicester, UK; 4 Department of Psychology, University of Nottingham, Nottingham, UK

**Keywords:** time perception, metacognition, ideal observer analysis, psychophysical methods

## Abstract

The ability of humans to identify and reproduce short time intervals (in the region of a second) may be affected by many factors ranging from the gender and personality of the individual observer, through the attentional state, to the precise spatiotemporal structure of the stimulus. The relative roles of these very different factors are a challenge to describe and define; several methodological approaches have been used to achieve this to varying degrees of success. Here we describe and model the results of a paradigm affording not only a first-order measurement of the perceived duration of an interval but also a second-order metacognitive judgement of perceived time. This approach, we argue, expands the form of the data generally collected in duration-judgements and allows more detailed comparison of psychophysical behavior to the underlying theory. We also describe a hierarchical Bayesian measurement model that performs a quantitative analysis of the trial-by-trial data calculating the variability of the temporal estimates and the metacognitive judgments allowing direct comparison between an actual and an ideal observer. We fit the model to data collected for judgements of 750 ms (bisecting 1500 ms) and 1500 ms (bisecting 3000 ms) intervals across three stimulus modalities (visual, audio, and audiovisual). This enhanced form of data on a given interval judgement and the ability to track its progression on a trial-by-trial basis offers a way of looking at the different roles that subject-based, task-based and stimulus-based factors have on the perception of time.

## Introduction

The perception of time from milliseconds to years is a cognitive capacity that fascinates from many different perspectives. Time, as a perceptual quantity, has some unique characteristics that also make it particularly challenging to measure and model. Of all the measurable properties of the world that we do somehow encode and experience, time is arguably the most distanced from its physical realization when considered in terms of its neural representation (Buonomano & Rovelli, 2022; Grondin, 2023; Gruber, Block, & Montemayor, 2022). Time is also unusual as a fundamental perceptual experience in that it can be derived from any sensory modality, although vision and audition are the most commonly investigated. Furthermore, the internal awareness of the duration of time passed, the metacognition of time, is critical for the maintenance of an ongoing awareness of, and involvement in, the external world and the segmentation of ongoing external events (Wahlheim, Eisenberg, Stawarczyk, & Zacks, 2022; Zacks, 2020; Zacks, Bezdek, & Cunningham, 2022). The loss of this (temporal) context is a major symptom of psychosis and seriously affects the ability of an individual to interact socially or otherwise with their environment (Bocker, Hijman, Kahn, & De Haan, 2000; Cohen & Dochert, 2005), and a compromised perception of time has been suggested as a diagnostic tool in childhood ADHD (Ptacek et al., 2019; Smith, Taylor, Rogers, Newman, & Rubia, 2002) . Alternatively, there are notable psychological states, such a creative “flow state,” trance or meditative states, where the loss of the sense of the passage of time is considered a positive and desired outcome (Csikszentmihalyi, Abuhamdeh, & Nakamura, 2005; Huxley, 1954). Time, and its passing, can be thought of as a thread through the ongoing narrative around which we construct our conscious lives, and although we all have our own “thread,” we also rely on substantial agreement about the passing of time to coexist functionally in the same intertwined reality.

The ability of subjects to identify and (re)produce brief temporal intervals is influenced by many factors whether stimulus, subject or task-based (Bruno, Ayhan, & Johnston, 2012; Chen & Yeh, 2009; Corcoran, Groot, Bruno, Johnston, & Cropper, 2018; Droit-Volet & Meck, 2007; Jazayeri & Shadlen, 2010; Mitani & Kashino, 2016; Ortega, Guzman-Martinez, Grabowecky, & Suzuki, 2014; Piras & Coull, 2011), with subject age and developmental stage attracting particular interest (Droit-Volet, Meck, & Penney, 2007; Droit-Volet & Wearden, 2001; Droit-Volet & Wearden, 2015; Droit-Volet, Wearden, & Zelanti, 2015; Pouthas, Droit, Jacquet, & Wearden, 1990). Several reviews have comprehensively outlined the current state of empirical and theoretical development in time perception (Block & Grondin, 2014; Bruno & Cicchini, 2016; Eagleman, 2008; Grondin, 2008; Grondin, 2010; Matthews, 2015; Matthews & Meck, 2014; Matthews & Meck, 2016; Meck & Ivry, 2016; Tsao, Yousefzadeh, Meck, Moser, & Moser, 2022); the aim here is not to repeat this information but to introduce and motivate what we believe is a useful method to approach the study of brief to moderate temporal intervals and the metacognition of that perceptual experience. We have used this approach to look at population differences in time perception and metacognition (Corcoran et al., 2018) but examine the methodology and data in a small-N psychophysical design in more detail here (Smith & Little, 2018), so we may develop a model of time perception and metacognition with a higher level of precision. To this end, we examine temporal bisection performance in healthy adults for subsecond (750 ms) and suprasecond (1500 ms) tasks within visual, audio, and audiovisual modalities and the ongoing metacognition of that performance.

### Ways of measuring time

Time is unusual^1^ as a sensory quantity in that it is not bound to one particular sensory input; from an experimental perspective, the two most common modalities chosen to generate the temporal stimulus are auditory and visual, but measurement of somatosensory time perception is also not uncommon (Ball, Arnold, & Yarrow, 2017; Tomassini, Gori, Burr, Sandini, & Morrone, 2011; Watanabe, Amemiya, Nishida, & Johnston, 2010). Although much of the work has taken a somewhat modality-agnostic approach and focused on the sense of time passed, there have also been notable studies focusing on the role of the sensory modality itself in the percept (Ball et al., 2017; Burr, Banks, & Morrone, 2009; Recanzone, 2003; van Wassenhove, Buonomano, Shimojo, & Shams, 2008).

**Figure 1.**
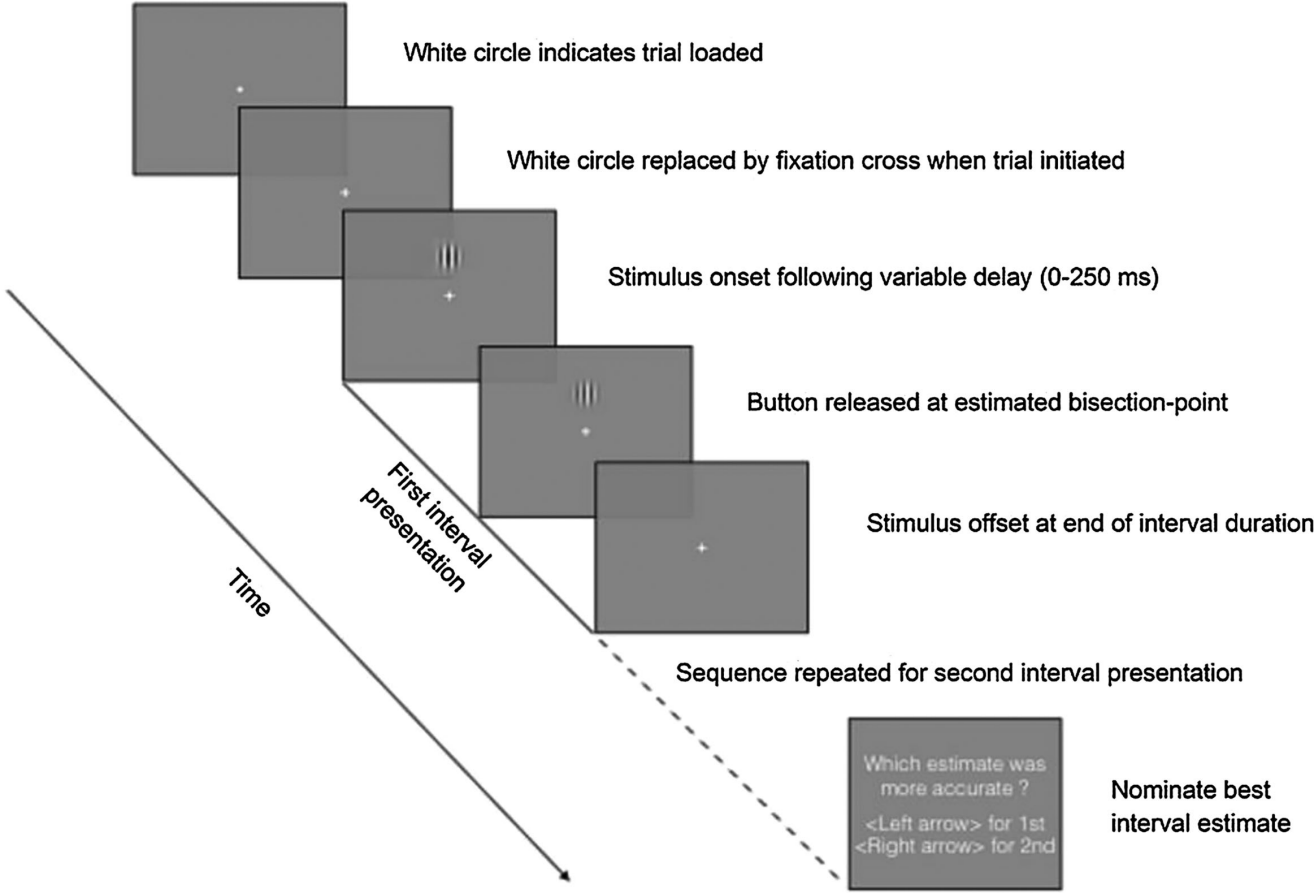
Schematic illustration of the modified temporal-bisection task trial. The stimulus is present for the full duration regardless of the estimated bisection-point. Auditory feedback was provided on whether the participant had correctly chosen the interval closest to the actual mid-point after the second sequence.

Grondin (2008); Grondin (2010) outlines four main methods of investigating time that have been traditionally distinguished within the literature: verbal-estimation, reproduction, production, and comparison. The final “comparison” category can be split into several subcategories (e.g., roving or reminder, single-stimulus, bisection), but this category overall is equivalent to standard psychophysical methods of measurement for any sensory capacity and tends to be favored in the experimental literature. Time perception from a sensory perspective is generally focused on the examination of durations up to a few seconds. These methods and approaches are neatly summarized in his Figure 1 (Grondin, 2010). The method examined in detail here falls under this broad “comparison” definition, and incorporates characteristics of both reproduction and bisection, with an additional metacognitive component.

### Bisection

Traditionally, in a standard temporal bisection paradigm, participants are given two “standard” or reference intervals, one shorter and one longer, which they are required to remember. They then judge a series of single “test” intervals to be closer to the shorter or to the longer standard interval, effectively bisecting the perceived difference between the two standards: the test interval that is judged to be closer to either standard half the time is the perceived bisection point (Kopec & Brody, 2010). For instance, if the shorter standard stimulus was one second and the longer standard was three seconds, the inter-standard interval is two seconds, and the veridical bisection point is also two seconds. The data collected from this paradigm allows construction of a psychometric function to identify the perceived midpoint in the interval between the two standards and the method is akin to the psychophysical Method of Single Stimuli (Macmillan & Creelman, 1991) used most effectively in measuring speed perception (McKee, Silverman, & Nakayama, 1986).

### Reproduction

Reproduction has, perhaps more simply, presented the participant with an interval, demarcated through whatever means (a continuous tone or light, or a pulse at the beginning and end of the interval is common) and then asked the participant to reproduce that interval through some means such as a button-press or finger-tap. The result is then expressed as a mean and variance in the reproduced interval compared to the test duration.

We have developed a combination of these two approaches which provides a behavioral measure that allows us to observe the participants’ estimate of perceived duration in a way that is not possible in the case of the perceptual comparisons generally used in the literature (Grondin, 2010). This paradigm also affords greater flexibility with the collected data, providing both a trial-by-trial estimate of a given test duration and a cumulative summary of the mean and variance of the estimate. Importantly, we also incorporate a metacognitive component whereby the participant is required to examine their own recent performance. This combined paradigm also reduces the reliance upon memory implicit in the standard bisection paradigm through the actual period to be bisected being shown each time the bisection/(re)production is enacted.

### Metacognition of time

Metacognition is defined as awareness and understanding of one's own thought processes (Fleming & Frith, 2014; Terrace & Metcalfe, 2005), and we suggest that the awareness of time-passed is crucial for interaction with the external world and the ongoing events defining that world (Franklin, Norman, Ranganath, Zacks, & Gershman, 2020; Zacks, 2020). In the current context, we refer to the awareness of one's own performance in the temporal task, with the intention of obtaining an objective measure of a participant's confidence in their performance (Caziot & Mamassian, 2021; de Gardelle, Le Corre, & Mamassian, 2016; Mamassian, 2016). We are interested in the potential to measure the (raw) ability of participants to bisect an interval and to also determine how well participants believed they performed in the task. Given that the percept of time can be so easily disrupted by many factors both commonplace and otherwise (Ayhan, Revina, Bruno, & Johnston, 2012; Brown & West, 1990; Cicchini & Morrone, 2009; Droit-Volet & Meck, 2007; Grommet et al., 2011; Maarseveen, Hogendoorn, Verstraten, & Paffen, 2018; Morgan, Giora, & Solomon, 2008), measuring metacognition in temporal tasks is an important extension to the current standards. We introduce this possibility here by asking the subjects to compare two consecutive time interval estimates and decide which they thought was the closer estimate to the actual midpoint of the test interval. Feedback can then be provided on the accuracy of this second-order judgement.

We recognize that in the context of our particular paradigm, the use of the term metacognition may differ from some other authors. This was a significant issue raised by both our reviewers and we respect their concerns. In short, we are interested in the insight that the subject has into their performance after completing two identical (bisection) tasks; to this end we ask them in which trial did they perform better once the tasks are complete. This means that their metacognitive judgement can be based on any or all information available to them for the period over which the two tasks were performed. It also means that all the information available to make the metacognitive decision is not available at the moment the perceptual (bisection) decision is made. We return to this issue in the discussion but raise it here to clarify our position at the outset.

### Confidence

Subjective confidence in a decision judgement can be argued to be an outcome of the metacognition related to that decision; this relationship could then provide a window to the underlying mechanisms of a given decision (reviewed in Mamassian, 2016). There have always been issues with measuring confidence in a judgement since it is, by definition, a subjective measure that is notoriously hard to standardize across observers. The common methodology of using a Likert scale to rate the degree of confidence in a given judgement is fraught with error (Caziot & Mamassian, 2021; Mamassian, 2016; Morgan, Mason, & Solomon, 1997) and significant efforts have been made to find a more effective way of measuring subjective confidence (Fleming & Lau, 2014; Maniscalco & Lau, 2012; Miyoshi, Sakamoto, & Nishida, 2022; Zizlsperger, Kümmel, & Haarmeier, 2016; Zizlsperger, Sauvigny, & Haarmeier, 2012; Zizlsperger, Sauvigny, Händel, & Haarmeier, 2014) . In this vein, we suggest that the forced-choice measurement introduced in this paradigm may be a direct way to overcome the problems associated with Likert scale confidence measures such as variable use of the full range between individuals, the underlying assumption of linearity across the range and variability both within and between subjects. We argue that the paradigm outlined here provides an objective and cumulative measure of confidence on a trial-by-trial basis. Furthermore, the unique structure of the data collected on the first- and second-order judgements described here allows a genuine ideal observer analysis of the data collected from each participant and each condition. Our approach is substantially similar to that of Caziot and Mamassian (2021), who argue that confidence should be considered a measure of internal consistency rather than external veridicality.

In summary, here we examine bisection performance in subsecond and suprasecond intervals coded by visual, audio or audiovisual stimuli and to see whether stimuli with two coherent sources of information (audiovisual) improves performance, and consistency in the knowledge of that performance, over that with a single source alone (visual or audio). We then analyze and model both aspects of that data, bisection and metacognition, using a hierarchical Bayesian approach, that facilitates comparison to an ideal observer.

## Method

### Participants

Participants (aged 23 to 53) were either the authors (SC, WLX) or recruited through word-of-mouth (N = 5; 3 of them were females). No participant reported any history of neurological or psychiatric disorder, nor was any individual taking ongoing medication. All participants had normal (or corrected-to-normal) vision and normal hearing. Experiments were approved by the Human Ethics Advisory Group at the University of Melbourne. Participants provided informed consent for participation and academic use of their (anonymous) data. Participants’ names were coded as JCZ, RXL, SC, SLY, WLX and were reimbursed for their time ($15/hour) with the exception of the authors.

### Apparatus and stimuli

Stimuli were developed using the Psychophysics Toolbox, Version 3 (Brainard, 1997) and MATLAB R2022a software package (The MathWorks Inc, Natick, MA). Stimuli were either visual (V), auditory (A), or a synchronized combination of the two (AV). A Mac Pro (early 2009, OS X El Capitan) computer was used to run the software while time-critical interval-judgement responses were collected via a calibrated Cedrus RB-530 (Cedrus Corporation, San Pedro, CA) response pad and metacognitive judgements via a (wired) Macintosh keyboard.

The visual stimulus was displayed on a SONY Trinitron Multiscan G520 monitor (resolution = 1600 × 1200 pixels, frame rate = 100 Hz, mean luminance = 40 cd/m^2^, CIE white point co-ordinates {x = 0.333, y = 0.377}) within a stationary circular envelope (diameter = 4°), the lower edge being located 4° above a central fixation spot. The audio was delivered through Sennheiser HD 25-mk2 headphones via a Roland UA-M10 external USB DAC.

The visual stimulus consisted of a vertically orientated sinusoidal luminance grating (spatial frequency = 1 cycle/°; 0.8 Michelson contrast) presented against a gray background at the mean luminance of the display (40 cd/m^2^). The auditory stimulus was a 480 Hz pure tone set at a moderate volume around 10 times detection threshold. The synchronized stimulus was the combination of both audio and visual stimuli from onset to offset. Both stimuli were presented in rectangular temporal envelopes as onset and offset were critical aspects of the stimuli and any temporal smoothing would affect their temporal definition. We are, however, mindful of the potential effect of any temporal artefacts introduced by this kind of envelope (Cropper & Badcock, 1994; Watson, Ahumada, & Farrell, 1986).

### Procedure

The experiment comprised the three stimulus conditions presented according to the testing schedule summarized in Table 1. Three sessions were carried out over three weeks. All sessions were completed in the same order for each participant so they were afforded the same opportunity to build up their internal representation of the duration to be bisected. We do acknowledge that using this ordered approach to data collection across subjects is not strictly best practice for repeated-measures designs, but we consider, given the aim of the experiment, it was appropriate in this case.

**Table 1.** Testing schedule. *Notes:* V, the visual stimulus condition; A, the auditory stimulus condition; AV, the audio-visual stimulus condition. There was a one-week interval between individual sessions.

Session number	Stimulus modality	Duration condition (msec)
Session 1	V	1500
		3000
Session 2	A	1500
		3000
Session 3	AV	1500
		3000

Each session took approximately three hours to complete, including a break of 15 to 30 minutes between the 1500 ms and the 3000 ms condition. There were periods of one week between each main session, and therefore between the visual, audio and audiovisual conditions, for each participant. The participants were also encouraged to take breaks between trial blocks if they wished and to report any undue fatigue. In terms of practice effects, these are explicitly built into, and a crucial part of, the paradigm; trial-by-trial analysis allows monitoring of the improvement in performance over time (e.g., see Figure 3). All subjects reported that the trial regimen was acceptable in terms of duration and structure and that they did not suffer from undue fatigue. The testing schedule is summarized in Table 1.

In each experimental session participants were tested with one stimulus condition (the visual, auditory, or audiovisual stimulus), which comprised 500 trials presented in blocks of 100 trials. Each stimulus condition was tested at two durations (1500 msec (bisection of 750 msec), 3000 msec (bisection of 1500 msec)).

#### The modified temporal-bisection paradigm

Each trial involved a temporal estimation phase and a metacognitive judgement phase. In the temporal estimation phase participants were required to make two interval-bisection estimates. In the metacognitive judgement phase, participants were required to identify which of the two, preceding interval-bisection estimates was, in their opinion, closest to the predefined target duration (the veridical bisection point). Participants were never shown the actual bisection interval, only the full test interval, learning the interval and its bisection point was part of the task. Each trial block (of 100 trials × 5 episodes) measured the same test interval to be bisected, allowing participants to build up an internal representation of the task interval. They were also told what the interval was they were trying to bisect at the outset.

A schematic representation of the generic trial structure is depicted in Figure 1 for a visual example. The stimulus may be of any modality providing it can be bisected in time. In the data discussed here, the stimulus was always presented for the duration to be bisected but we have also used two pulses, one at the beginning and one at the end of the interval, as a modification of the single continuous stimulus, and varied the modality of the stimulus between the intervals to be compared (Cropper, Kendrick, Goodbourn, Bruno, & Johnston, 2018).

All trials consisted of two identical intervals (one of which is represented in Figure 1), each being demarcated through the continuous presentation of the test stimulus; in the example illustrated in Figure 1, the temporal cue was solely visual. As indicated above, the participant was told the interval duration that was to be used for the entire block of trials and that they were required to estimate half of this interval, bisecting the period that the stimulus was present on the screen. The first interval was initiated when the participant depressed the response pad button. To avoid the use of rhythm in the task (which we found, through pilot testing, made the task trivial), a random delay, ranging from 0 to 250 msec, was inserted after the button press and prior to the appearance of the stimulus. Upon appearance of the stimulus, the participant maintained button depression until the perceived interval midpoint, when they released the button. The stimulus remained on-screen for the full duration of the interval. This procedure was then repeated for the second interval estimate following a 500 msec inter-stimulus interval.

After making a pair of bisection-point estimates, and a further 500ms interval, participants were prompted to make a two-alternative forced-choice response via the keyboard arrow keys to indicate which of their estimates they deemed closest to the target duration (i.e., their “best” estimate). The choice remained on screen until the decision was made (Figure 1). This retrospective judgement of the two bisection intervals constitutes the temporal metacognition component of the task. A brief tone indicated whether the participant's choice had been the interval closest to the veridical bisection point or not. Overall, this paradigm therefore contains a first-order judgment, which is a combination of temporal-bisection and interval-reproduction, followed by a second-order forced-choice judgment of one's own accuracy (Caziot & Mamassian, 2021; Mamassian, 2016).

The exact strategy used by each observer to do the task was up to them, as it invariably is in all psychophysics. It is our intention that the subjects perform the task as best they can, however they can, and use the feedback given, whether explicitly or implicitly through their own monitoring, to improve on their performance in the subsequent trial-pair. We consider that they are most likely to use all evidence available to them, which implies that the subject can use both the first part and the second part of either interval (i.e., pre- and post-button-release) as evidence about whether they made an accurate assessment of the mid-point. The noise associated with either of these judgements (pre- or post-release) is the same. The substantive point is that for the metacognitive component of the task the observer must make a judgment about performance on both trials taken as a whole, wherever the information they use on each trial to come that decision may come from. For instance, for a total duration of 1500 msec, they could use the pre-release interval they believed to be closest to 750 msec, the post-response interval closest to 750 msec, the entire interval in which pre- and post-release interval were most alike, or the best indicator of all these three sources of evidence on any given trial. We are agnostic about the source of information used but interested in what this information tells the observer about their performance.

### Treatment and analysis of the data

One of the benefits of the modified bisection paradigm is that the data collected can be examined both as a trial-by-trial analysis of the subject performance and, more traditionally, as an expression of performance over the whole experimental condition. Data analysis and modeling was performed in Matlab (R2022a), JASP (Version 0.18), (JASP Team (2023)) and R (v4.3.1).

#### Basic raw data and sorting of individual responses

**Figure 2.**
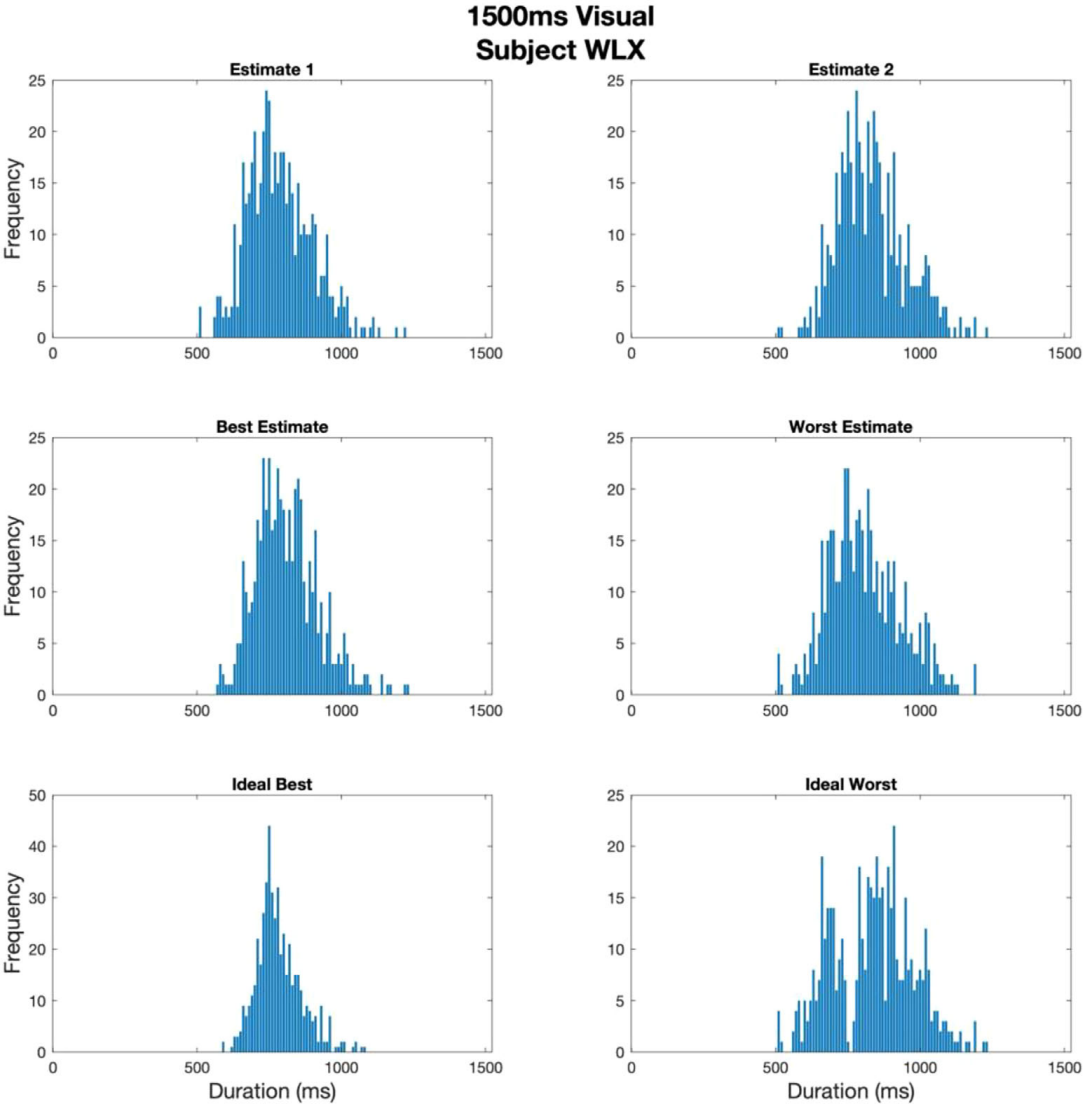
Histogram plots using 150 bins from estimates of zero to the total stimulus duration for one observer (WLX) in the visual 1500 msec stimulus duration condition. To characterize observers’ second-order responding, the trial-by-trial data were redistributed according to observers’ best estimate judgements, to gain mean and variance values of subjective best estimates, and by complement, subjective worst estimates, across trials within a condition. The data were also redistributed according to the ideal best and ideal worst estimates to gain the mean and variance in the case of ideal observer performance (i.e., the estimate distributions expected if observers had perfect insight into their own performance). Note the increased y-axis range in the Ideal Best condition.

**Figure 3.**
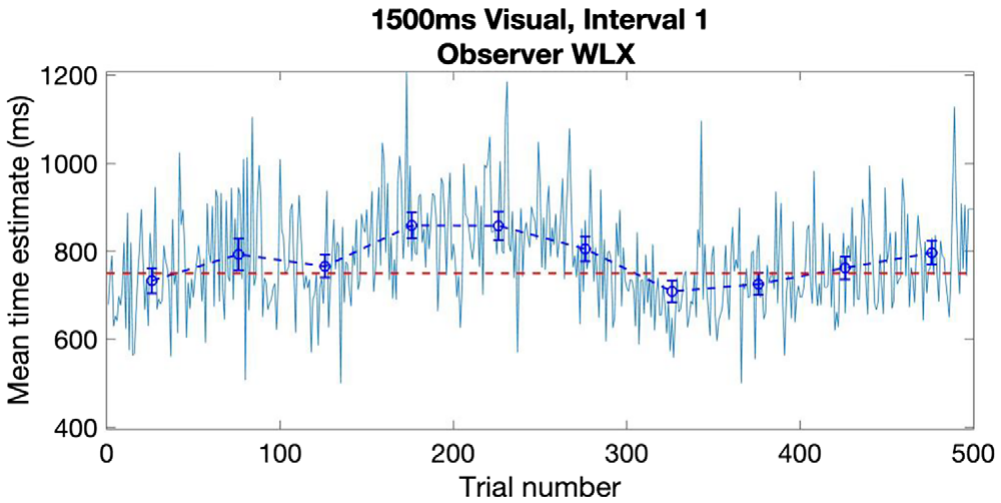
Raw trial-series of interval 1 for observer WLX (teal line), for the visual 1500 ms stimulus duration condition. The veridical bisection point at 750 msec is shown as a horizontal red dashed line. Binned means with 95% CI shown in royal blue, connected by dashed lines.

Example raw data for one observer (WLX) are shown in Figure 2 as frequency histograms plotting the estimated half-point of the 1500 msec interval for 500 trials. The data are presented in three different formats, sorted by rows on the figure. The first row plots, as a histogram, the bisection data for the first and second interval estimates respectively. The second row re-sorts the data into the perceived best (left-hand side) and perceived worst of each pair. The third row plots the actual best and worst estimates (i.e., the ideal observer response). Each of the distributions has a mean and variance that can be used to summarize performance across all the trials and the trial-by-trial data can be analyzed as a time-series for a given interval (Figure 3). The individual trial estimates, followed by each pairwise analysis of performance, allow a subjective ideal Observer analysis for each subject and each set of conditions. The bimodal pattern of the “ideal worst” response is somewhat exaggerated in this example (real) dataset but is a direct and predictable product of the data-sorting algorithm and depends on the trial-by-trial values of the particular dataset, which are influenced by both subject and condition and also by the similarity between the two bisection estimates of each trial pair. For instance, the “ideal worst” response will always have the estimate further from the mean of any given pair creating a distribution containing the tails of the original population, the “ideal best” response set receiving the data closest to the mean. However, this distinction becomes less clear as the two estimates approach each other in value. Grouping the data in this way allows the difference in variance between the best and worst distributions to be used as a measure of the knowledge of the observer of their own performance (Corcoran et al., 2018). Although the variance is not an ideal measure of a (potentially) bimodal distribution, as noted by one of our reviewers, it does capture the difference in the upper and lower bounds of the data set to a first approximation and we consider it to be a reasonable way of quantifying this difference in the current instance for the subsequent calculation of the Metacognitive Index (MCI; see below).

#### Analysis of performance over the duration of the experiment

Figure 3 plots the performance on a trial-by-trial basis for a single interval of the pair (Interval 1 in this case—summarized in the top left graph in Figure 2) over the entire 500 trials for one subject (WLX). The individual bisection estimate (y axis) is plotted against the trial number (x axis). The binned means (bins of 50 trials, data points in middle of bin), with 95% confidence intervals superimposed on the trial-by-trial data. The benefit of looking at the data this way is that one can see how performance changes over the duration of the experiment.

**Figure 4.**
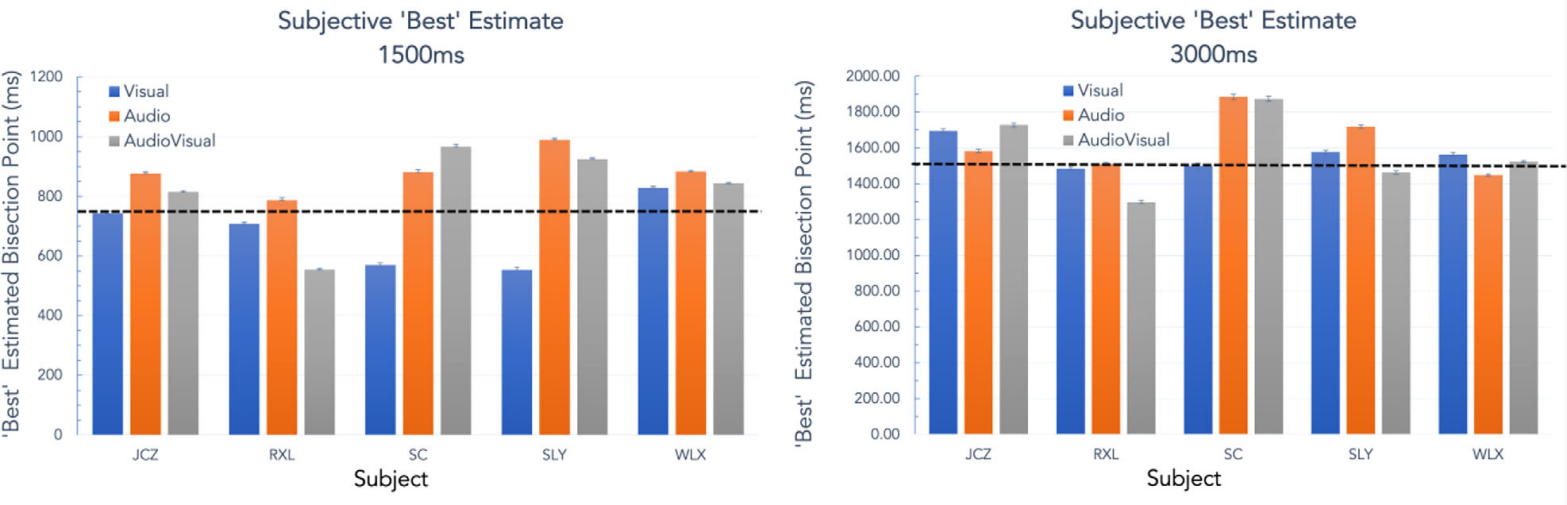
“Best” estimate of the interval bisection plotted for five subjects across three conditions (Visual, Audio and Audiovisual). Error bars are ± SEM, and the dotted line indicates the veridical bisection point.

This trial-by-trial approach to the analysis can be extended to examine how previous trial performance affects the current trial either globally by curve-fitting the data or more locally by conducting an autocorrelation of the data. Each of these approaches offers some possibility of examining how the internal representation of the target interval builds up for the participant over the period and how recent performance affects the current decision. It also gives a clear indication of any effects of practice/perceptual learning or fatigue over multiple (5 × 100 trials) sessions. Figure 3 is typical of all our subjects in that after an initial “guessing” phase the duration estimate approaches the veridical bisection point, albeit with an overshoot and return in this case. Overall mean performance then settles to remain approximately constant across the 500 trials. The form of the trial-by-trial data in conjunction with subjective reports suggests that perceptual learning saturates after about 25 to 50 trials and the testing regime does not exhaust the subject. Although we adopt a purely descriptive approach in the current work, it is worth noting that initial examination of our data suggests there were no observable differences in the trial-by trial pattern of data between each session (i.e., each week), indicating that the learning phase was limited to within each session rather than across sessions. This observation is broadly consistent with recent work examining short- and long-term perceptual learning in a variety of tasks ranging from contrast discrimination to facial recognition (Yang et al., 2022). Within-session (40 minutes to an hour) learning elicited far greater effects than between session (23.6 hours) learning for all tasks, most of which showed no significant between-session learning (Yang et al., 2022; Figure 4). Given our between-session period was a week, we are confident this is unlikely to be a factor in our results.

#### Summary analysis: Re-sorted data

As implied in Figure 2, a summary analysis of performance is perhaps the most obvious and common way of looking at the data. The mean and variance of the two intervals (Row 1 of Figure 2) give the accuracy and precision respectively of the two bisection estimates and are the standard way of representing this kind of data, providing a means of comparing performance in this paradigm to others in the literature. This approach also allows the coefficient of the variation (i.e. standard deviation of estimated duration/mean estimated duration) to be used to express the adherence, or otherwise, to Weber's law, which predicts a constant coefficient across different bisection periods and is an enduring critical component of the scalar expectancy theory of time (Corcoran et al., 2018; Grondin, 2014; Wearden & Lejeune, 2008) .

#### Metacognition, feedback, and ideal observer analysis

Re-sorting the individual interval 1 and 2 trials into perceived best and perceived worst, and ideal best and ideal worst, gives some insight into how effectively the individual subjects in each condition can access and reflect upon their own performance during the task. We expect (and observe (Corcoran et al., 2018)) that the difference between variance of the estimates will be minimal in the Interval 1/2 condition and maximal between ideal best/worst. The difference in the subjective best/worst case will be somewhere between these two extremes and this difference can be used to quantify the degree of knowledge each subject had of their own performance in the bisection/reproduction task. To avoid concerns about the best function to fit to the collected data, and to be consistent with the subsequent modeling, we take a purely descriptive approach to the raw data analysis and calculate the arithmetic mean and variance directly in Matlab using Mean() and Var() functions.

One way of summarizing the metacognitive performance of the subject for a given set of conditions is to calculate a ratio of the subjective and ideal variance ratios as follows:

(1) $$\mathrm{MCI}=\left(\frac{V_{i.\mathrm{best}}}{V_{i.\mathrm{worst}}}\right)\;/\;\left(\frac{V_{s.\mathrm{best}}}{V_{s.\mathrm{worst}}}\right)$$

where the MCI is the Metacognitive Index for a given subject and condition, V is the variance of the ideal or subjective best/worst interval (indicated by the subscript). If the subject has little knowledge of their own performance, then the variance of the ‘best’ and ‘worst’ intervals will be substantially the same as that of the first and second intervals (top row, Figure 2) and the ‘subjective’ denominator of Equation 1 (Vs. best Vs. worst ) will tend toward a value of 1. The overall MCI will be the “ideal” numerator (Vi. best Vi. worst ), tending toward, but not reaching, zero, divided by the subjective denominator (approximating 1), resulting in a value close to zero. Alternatively, if the subject has perfect (ideal) knowledge of their own performance then both (ideal) numerator (Vi. best Vi. worst ) and (subjective) denominator (Vs. best Vs. worst ) of Equation 1 will be the same, giving an overall result of 1. Although the extreme values of 0 and 1 for the MCI are highly unlikely given the variances are of actual subjective data, we argue that this is a reasonable way to compare metacognitive performance between conditions and observers. Although there are some instances where the “ideal worst” distribution is bimodal as in the example given in Figure 2, we are primarily interested in the tails of the distribution, which are well represented by the variance.

### Empirical results

**Figure 5.**
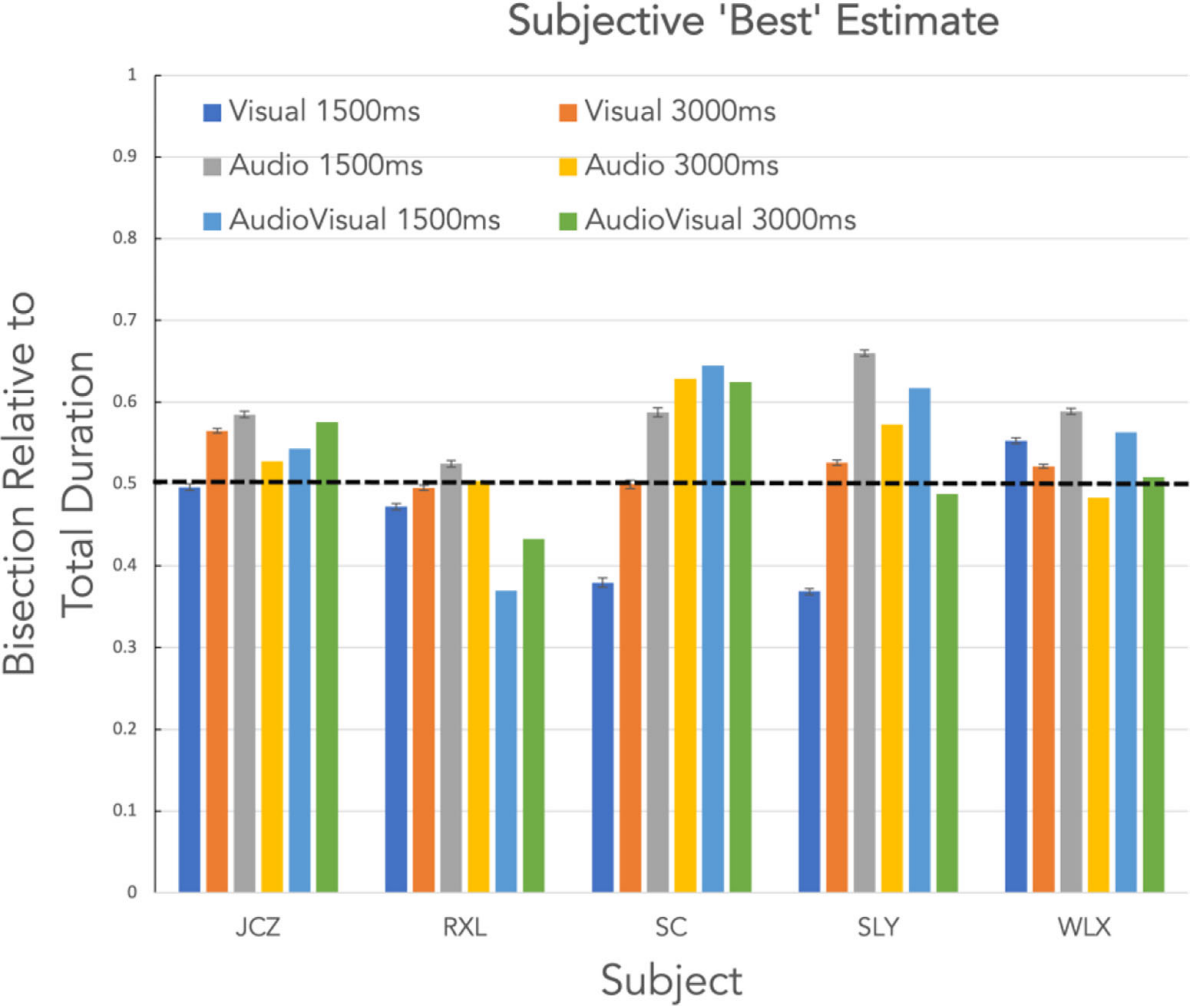
“Best” estimate of the interval bisection plotted relative to the total duration for the five subjects across conditions (Visual, Audio, and Audiovisual, 1500 and 3000 msec). Error bars are ± SEM, and the dotted line indicates the veridical bisection point.

Subjective “best” estimates for the bisection of the two presentation intervals (1500 ms and 3000 ms) are presented as bar-charts in Figure 4 for five observers across each of the three conditions (visual [V], audio [A], and audiovisual [AV]). There is some individual difference in performance, as expected (Corcoran et al., 2018), but all subjects do relatively well in the task in all conditions and also show a good degree of consistency with small error bars (±1 SEM). Overall performance appeared closer to the veridical bisection point in the longer duration condition. The trend for the visual condition to be underestimated relative to the veridical, compared to an overestimated audio or audiovisual condition seen at the short duration, all but disappears at the longer duration. Figure 5 replots this data as the bisection point in each condition as a fraction of the total duration where the relative bisection of 0.5 (dotted horizontal line in Figure 4) indicates veridical performance.

**Figure 6.**
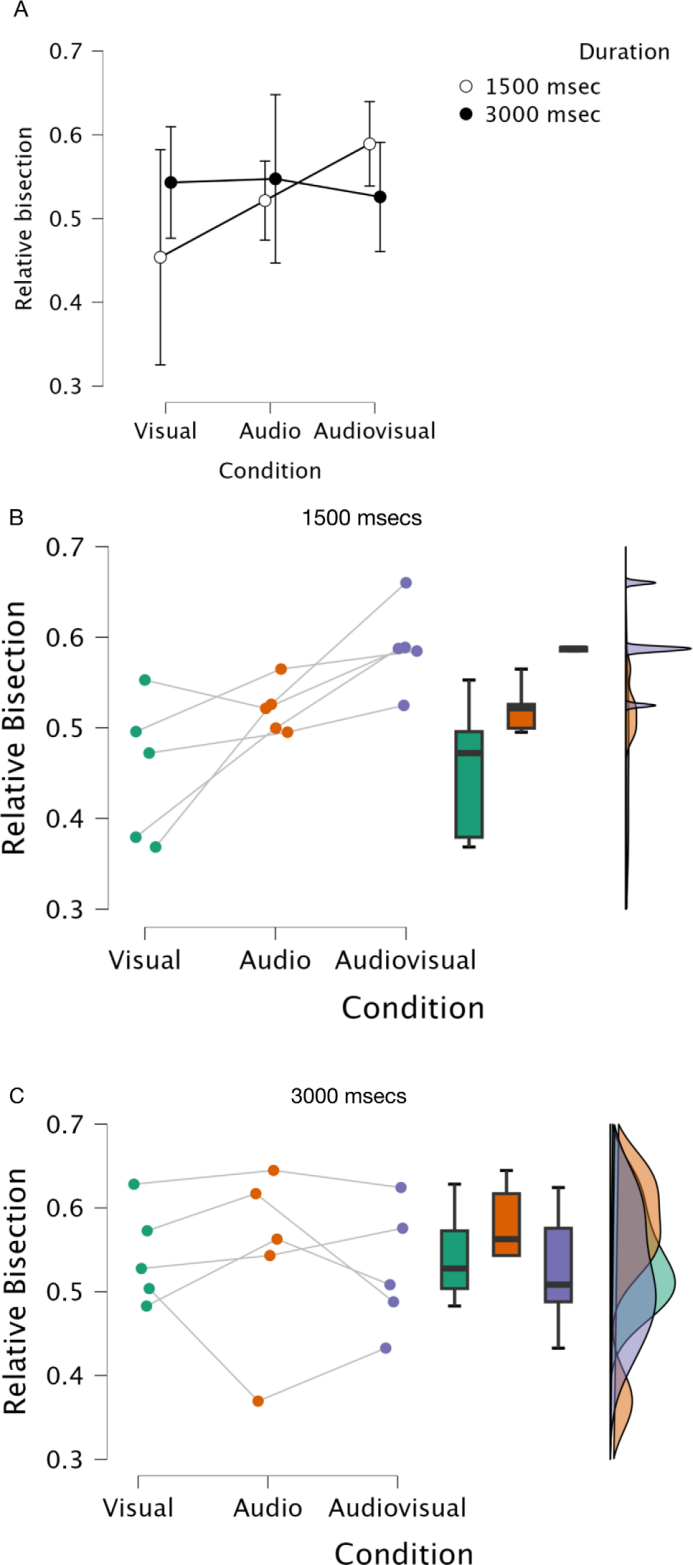
Results of Bayesian repeated measures ANOVA for relative bisection performance with stimulus “condition” and “duration” as factors for the five subjects shown in Figure 5. (**A**) Overall means; (**B**) By subject 1500 msec; (**C**) By subject 3000 msec. A relative bisection value of 0.5 (y axis) indicates veridical performance. Error bars in (**A**) are 95% credible intervals. In b and c, individual data points are plotted on the left-hand side for each subject and condition. The boxplots in the center give the median value (bold horizontal line), the interquartile range between the twenty-fifth and seventy-fifth quartile (colored bar). The “whiskers” indicate the minimum and maximum limits (excluding outliers – individual data points visible in the main line plot). Density plots on the right-hand side give the overall distribution of the data across the range (Allen et al., 2012). Output values for the calculations are given in Table 2.

To provide some quantitative analysis of the basic bisection performance, albeit for only 5 subjects, Figure 6 plots the results of a Bayesian repeated-measures analysis of variance (ANOVA) for the subjective “best” performance for the five subjects with the conditions and duration as factors in the analysis (Overall means [Figure 6A]; By subject 1500 msec [Figure 6B]; By subject 3000 msec [Figure 6C]); values for the analyses are given in Table 2.^2^ Given the few subjects used in the small-N design, any suggestion of within-subject effects are very small, and we acknowledge this. However, bearing in mind the meaning of Bayes factors given below,^2^ we interpret our data with caution. The intersection of the two duration lines in Figure 6A suggests an interaction of the condition and duration factors and this is timidly supported by the model of best fit being the “full” model in Table 2 (BF_10_ = 2.725). The greater effect of stimulus modality condition at the short duration on bisection performance than the longer one is made illustrated in Figure 6A by the positive slope in the 1500 msec curve compared to the flatter 3000ms curve. Individual duration data is shown by Raincloud plots (Allen et al., 2012; JASP_Team, 2023) in Figures 6B and 6C (see figure legend for description of the plots). Given a main point of interest in the empirical work here is the effect of stimulus modality, it is not unreasonable to examine each duration independently as a “planned comparison.” When we do this we see a much stronger (i.e., BF >10) effect of condition at the short duration only (1500 msec BF_10_ = 10.686) and no effect at 3000ms (BF_10_ = 0.356) (see Appendix A for more detail and post-hoc analyses). We note at this stage that the modeling section to follow is essentially a detailed hierarchical analysis of the raw behavioral data and so will offer an alternate analysis and validation of this data.

**Table 2.** Bayesian RM-ANOVA model comparison—“Best” bisection. *Notes:* All models include subject and random slopes for all repeated-measures factors.

Models	P(M)	P(M|data)	BF_M_	BF_10_	Error %
Null model (incl. subject and random slopes)	0.200	0.190	0.938	1.000	
Condition + Duration + Condition ✻ Duration	0.200	0.518	4.292	2.725	1.173
Condition	0.200	0.123	0.561	0.647	0.848
Duration	0.200	0.103	0.458	0.541	2.132
Condition + Duration	0.200	0.067	0.286	0.351	1.368

**Figure 7.**
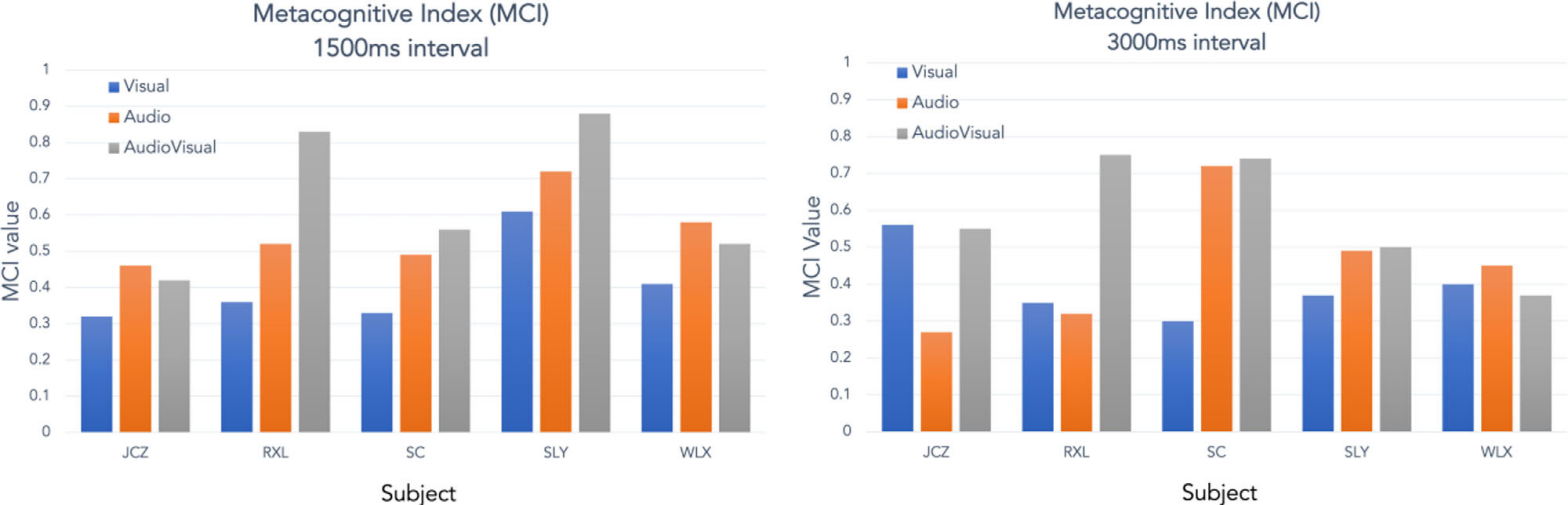
MCI for each subject and condition, the bisection data for which is plotted in Figure 4.

Figure 7 plots the metacognitive index (MCI) for each subject and condition shown in Figure 5. The closer the MCI value is to 1, the better the knowledge the subject has of their own performance in each trial-pair, and the closer to ideal performance. Although there are clearly individual differences in metacognition as there are in the interval estimation, there is a tendency for the audiovisual condition to give a higher MCI than either modality alone in three of the subjects (RXL, SC & SLY) although not with subjects JCZ & WLX.

**Figure 8.**
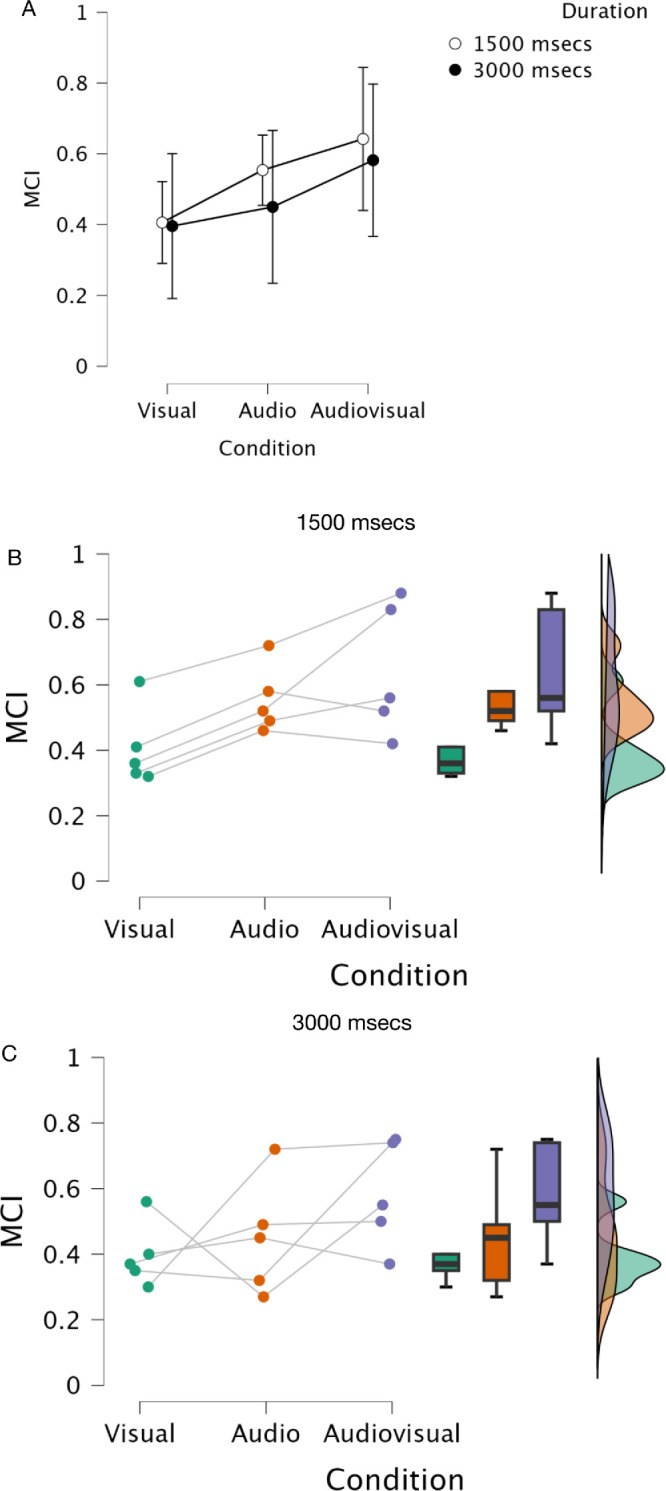
Results of Bayesian Repeated Measures ANOVA for MCI performance with stimulus “condition” and “duration” as factors for the five subjects shown in Figure 7. (**A**) Overall means; (**B**) By subject 1500 msec; (**C**) By subject 3000 msec. Error bars are 95% credible intervals. Output values for the calculation are given in Table 3. For description of the Raincloud plots see Figure 6.

A Bayesian repeated-measures ANOVA with condition and duration as factors was again calculated on the individual means from Figure 7. The group means are plotted in Figure 8a, which makes the trend across modalities clearer whereby the insight the subject has into their performance improves from visual through audio to audiovisual. The actual insight into performance appears to be slightly reduced for longer durations and there is no interaction between factors evident in the graph. The preferred model is condition alone (Table 3: BF_10_ = 2.415), of which we make barely a mention, with no interaction between factors (full model BF_10_ = 0.656). Consideration of the effect of condition independently at each duration gives a slightly more encouraging view suggesting a moderate effect at only the shorter duration: 1500 msec: BF_10_ = 7.568; 3000 msec: BF_10_ = 0.993 (For these and post hoc analyses, see Appendix A). Individual subject results from the ANOVA are plotted in Figures 8b and c for 1500 msec and 3000 msec, respectively, with the overall output values shown in Table 3.

**Table 3.** Bayesian RM-ANOVA model comparison—MCI. *Notes:* All models include subject, and random slopes for all repeated measures factors.

Models	P(M)	P(M|data)	BF_M_	BF_10_	Error %
Null model (incl. subject and random slopes)	0.200	0.161	0.767	1.000	
Condition	0.200	0.388	2.540	2.415	3.389
Condition + Duration	0.200	0.242	1.279	1.507	1.521
Condition + Duration + Condition ✻ Duration	0.200	0.106	0.472	0.656	1.589
Duration	0.200	0.103	0.459	0.640	1.139

#### Interim summary of empirical data

Given our caveats and caution above, the general suggestion from the empirical data is that in terms of bisection accuracy, there is no difference between the visual, audio, or audiovisual conditions at the 3000 msec presentation duration (i.e., on average), they are all slightly overestimated compared to veridical but equivalent to one another. In the 1500 msec duration, the audio condition is more accurate than the other two conditions; there is a modest trend for the visual condition to undershoot the bisection point and the audiovisual condition to overshoot. When considering the MCI data, which sorts the bisection data on the basis of the metacognitive decision, the results show that there is an increase toward ideal performance (a higher MCI) from the visual through audio to the audiovisual condition and this is overall slightly better at the shorter duration, but again with only five subjects these data only constitute a bare whisper of an effect.

### Hierarchical Bayesian model of bisection estimates and metacognitive accuracy

In the purely empirical analysis above, the MCI accounts for the metacognitive decision and bisection data simultaneously by using the metacognitive judgment as a basis for informing the sorting of the bisection data. In this section, we have taken an alternative analytical approach by modeling the metacognitive decision directly while simultaneously modeling the bisection judgments. This model is psychological in the sense that we consider the computational decision problem faced by the observer during the bisection and metacognitive stages of each trial (Marr, 1982) and we model each decision as a probabilistic choice with the latent parameters of decision distributions estimated in a hierarchical Bayesian manner (Lee & Wagenmakers, 2014). The benefit of this hierarchical analysis is each observer informs the group-level distribution, which “shrinks” estimates of subject-level parameters toward the group average (Britten et al., 2021; Davis-Stober, Dana, & Rouder, 2018; Davis-Stober, Dana, & Rouder, 2019; Gelman et al., 2004) allowing for more accurate estimates at the group level. Since we estimate posterior distributions over all quantities associated with the experiment for each participant, we can show that the model can predict the observed interval bisections estimates and the derived MCI data from each condition, while unpacking this latter measure into an alternative demonstration of ideal observer behavior.

We modeled the metacognitive decision on each trial, *r_i_*, as a Bernoulli distribution with a parameter, *p_i_*, indicating the probability that interval 1 was chosen as the more accurate interval on trial i. This parameter was derived from a comparison of the perceived error in each interval as follows:

Let Δ_1i_ and Δ_2i_ be the error in intervals 1 and 2, respectively, on trial i. That is:

$$\begin{array}{c} \Delta_{1i}=\left|I_{1}-\varphi \right|\\ \Delta_{2i}=\left|I_{2}-\varphi \right| \end{array}$$

where *I*_1_ and *I*_2_ are the bisections made by the participant on intervals 1 and 2, respectively, and φ indicates the subjective midpoint of the interval. The subjective midpoint of each of the visual, audio and audiovisual conditions is estimated around the subjective midpoint estimate across all conditions, which is itself assumed to be, as a reasonable starting assumption, normally distributed around the true midpoint. We model the bisection error by assuming that the observed interval estimates *I*_1_ and *I*_2_ are normally distributed with an observer-specific standard deviation (which is unique to each condition—modality × duration—and interval).

The model assumes that the participant chooses the option with the smaller perceived error. To implement this, we find the difference between the error in each interval: $d_{\Delta }=\Delta_{1}-\Delta_{2}$. (Here, we've suppressed indexing by trial for simplicity). If the difference is negative, then the error in the first interval is smaller, and the participant should choose the first interval. If the difference is positive, then the error in the second interval is smaller, and the participant should choose the second interval.

We assume that this choice occurs only probabilistically and that the difference between the error estimates is normally distributed around the true error difference, with a standard deviation representing the accuracy of the error estimation.

$${\hat{d}}_{\Delta }∼Normal\left(d_{\Delta },\sigma_{m}\right)$$

In the ideal observer model, the standard deviation would approach zero and consequently the representation of the error would approach the true error. Hence, the estimate of the standard deviation gives us a direct comparison to the ideal observer.

*p*_i_, which determines the Bernoulli outcome of the metacognitive decision, then equals the integral from the distribution of ${\hat{d}}_{\Delta }$between − ∞ and 0.

$$p_{i}\;=\int_{-\infty }^{0}P\left({\hat{d}}_{\Delta }|\;d_{\Delta },\sigma_{d}\right)$$

**Figure 9.**
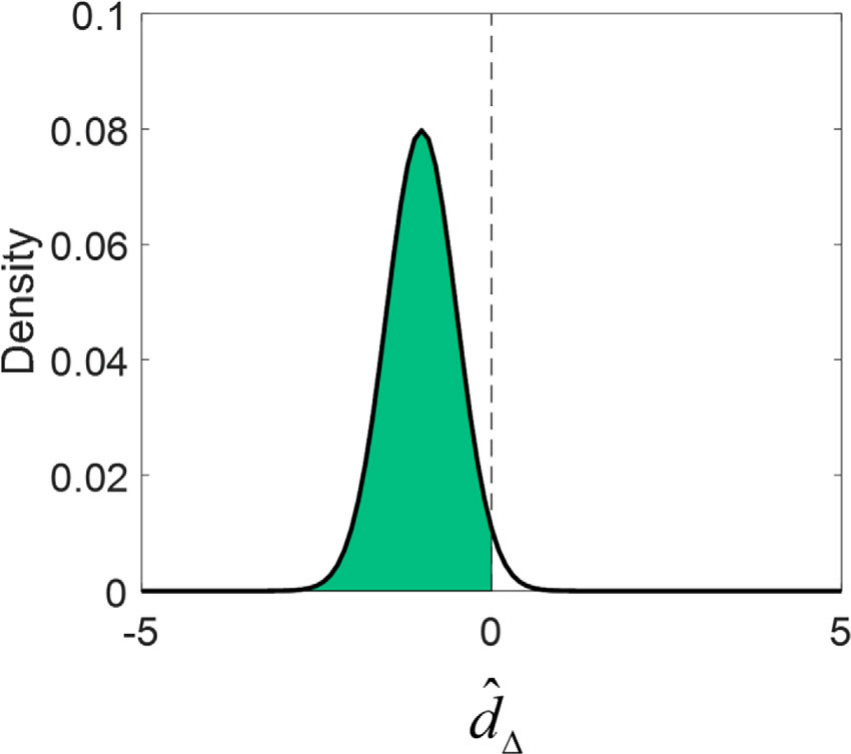
Illustration of how the probability of responding with interval 1 on a given trial is derived by integrating the distribution over the perceived difference in error.

When the error in interval 1 is smaller than in interval 2, the distribution will have most of its area in the negative region, and hence, when variance is small, *p*_i_ will be close to 1 (see Figure 9). When the error in interval 2 is smaller than in interval 1, the distribution will have most of its area in the positive region and *p*_i_ will be near 0 (i.e., interval 2 will be preferred). As the standard deviation approaches 0, the model will choose the interval with less error more accurately.

We estimated the standard deviations, σ_*I*1_, σ_*I*2_, and σ_*d*_ (along with the standard deviations for the midpoint estimates, σ_Φ_, σ_*Mid*_ ), hierarchically. We implemented the model in JAGS (Plummer, 2003) with Matlab and matjags (Steyvers, 2011) using two chains with a burn-in period of 2000 and a sampling period of 5000 samples, thinning every twentieth sample. We assumed a group-level distribution over the standard deviations for the visual, audio and audiovisual conditions. JAGS specifies the spread of the normal distribution in terms of precision (i.e., $1/\sigma^{2}$); the prior distributions for each precision parameter were uniform from 0 to 100. To capture the repeated-measures design, we assumed that the subject-level precision parameters were sampled from a multivariate normal distribution with a shared variance-covariance parameter and a subject-specific correlation parameter that captured how correlated the variance estimates were across the three conditions. All precision estimates were transformed to standard deviations for ease of interpretation; a smaller standard deviation indicating greater precision in the judgement.

In a nutshell, group-level standard deviation estimates for the visual, audio and audiovisual conditions measure the variability of the interval estimation and the metacognitive accuracy. Values near 0 indicate more accurate (veridical) estimates. At the group-level, these parameter estimates allow inference as to the difference between each modality and the combined audiovisual condition. The subject-level parameters indicate the performance of different individuals and allow an assessment of individual differences in bisection and metacognitive judgment accuracy. Because the estimates are posterior distributions of the parameters given the data, we can make inferences about the difference in value by directly comparing these distributions and examining their overlap. We can also use the 95% credible interval estimates as an inferential statistic. The credible interval is the region of the posterior distribution containing 95% of the posterior probability. A more flexible version of the credible interval, in that it deals with skewed distributions better, is the highest density interval (HDI), and it is this we calculate in the model (Kruschke & Liddell, 2018).

**Figure 10.**
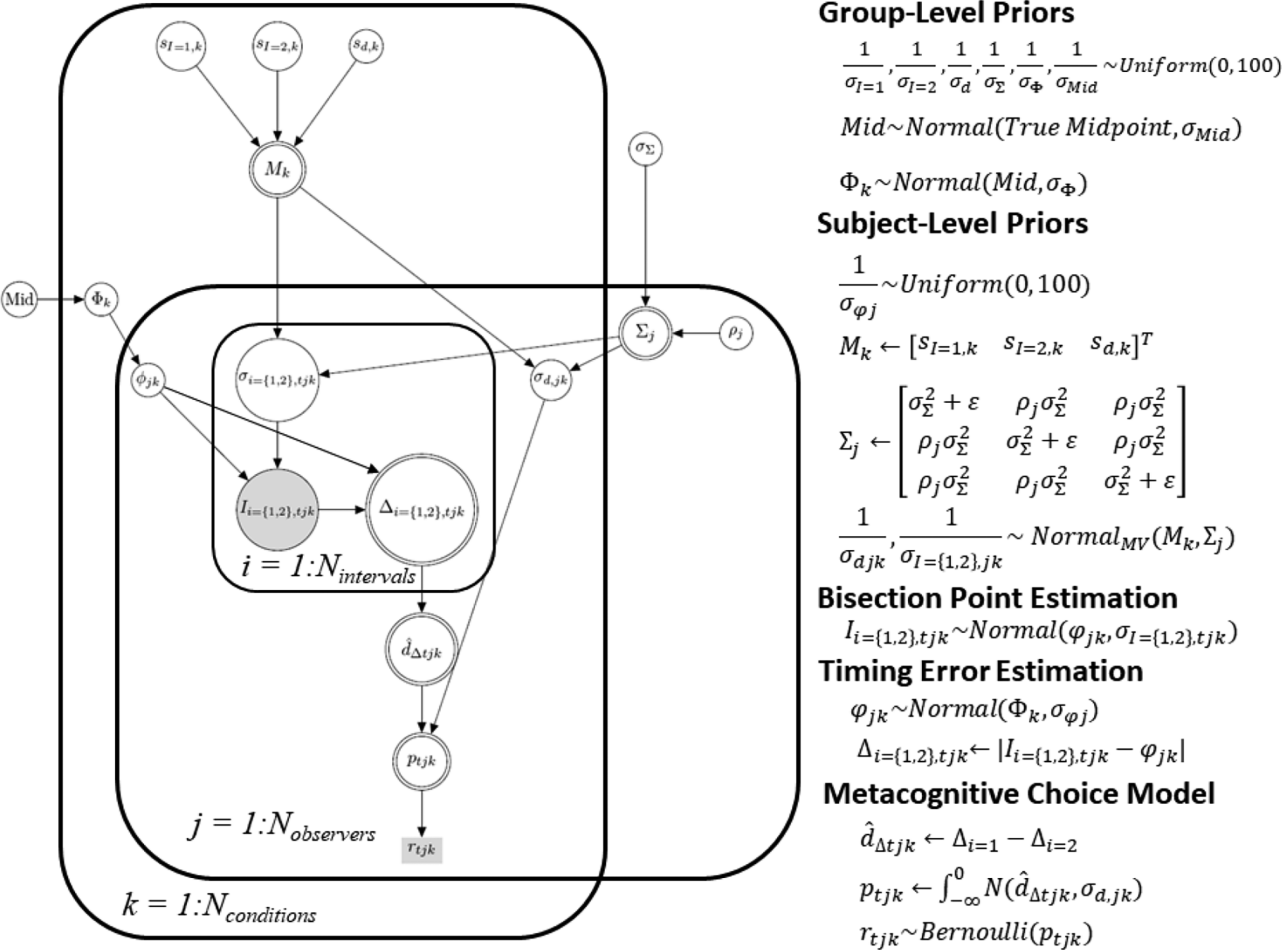
Illustration of the model as a directed graph (see e.g., Lee & Wagenmakers, 2014). Shaded boxes illustrate observed discrete variables, double circled nodes indicate derived variables, and white circles indicate continuous latent variables. The likelihood and prior distributions are listed to the right of the graphical model. Variables are defined in text; ε = 0.1 to ensure a positive semi-definite covariance matrix.

**Figure 11.**
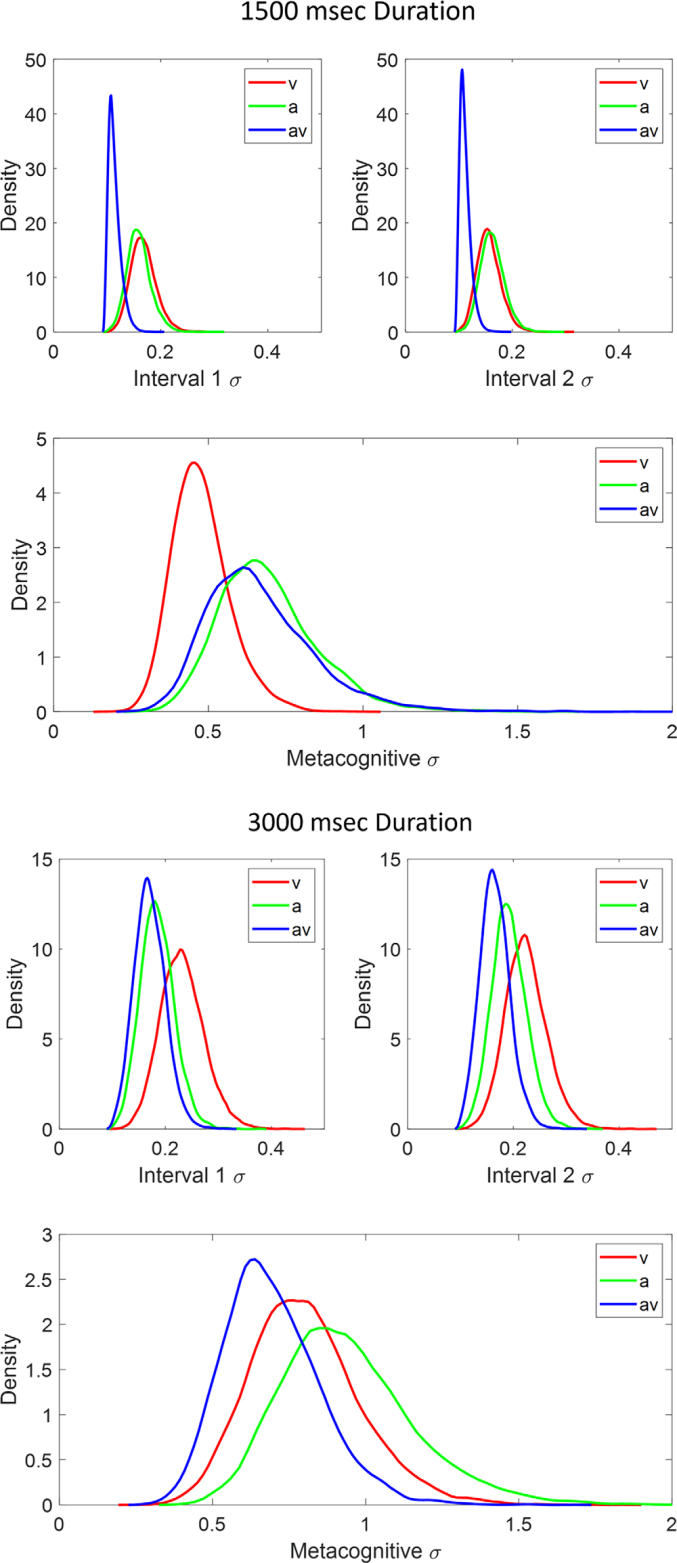
Posterior densities for the subjective interval midpoint standard deviations and subsequent metacognitive decision standard deviations in the V, A, and AV conditions for 1500 and 3000 msec durations; upper and lower panel, respectively.

Figure 10 shows the graphical model used to estimate parameter posteriors for each duration condition. Of note is that the latent parameter estimates are constrained by *all* of the data, including the bisection estimates for each interval and the metacognitive choice for each trial and each subject from each condition. All chains showed good convergence (maximum R-hat = 1.0002; Gelman et al., 2004). Figure 11 shows the posterior density estimates for the group level parameters. Specifically, the top two panels for each condition show the standard deviation of the subjective interval estimation while the bottom panel (for each condition) shows the standard deviation of the normal distribution for the metacognitive decision. The metacognitive decision is driven by the standard deviation estimate shown in this lower panel along with the difference in the perceived error between interval 1 and interval 2 (i.e., ${\hat{d}}_{\Delta }$ as described above).

The interval bisection standard deviation estimates have roughly the same posterior regardless of whether the stimulus was presented visually, auditorily or as a combined audiovisual signal, though in the 1500 msec condition, the audiovisual condition had more posterior density over smaller estimates than in the visual or audio conditions alone. In the 3000 msec condition, the posterior distributions are more similar. In sum, the estimates have roughly equivalent precision between modality and duration conditions (Corcoran et al., 2018; Grondin, 2014; Mitani & Kashino, 2016; Wearden & Lejeune, 2008). It appears the benefit of having a redundant audiovisual signal (i.e., the same information from two sources) is less prominent when the duration to be estimated is longer. Comparing the posterior estimates across the top two panels, there does not appear to be any difference between the estimates for interval 1 and interval 2.

The variance of the metacognitive judgement, which determines the accuracy of the judgement, varied depending on whether the interval was 1500 msec or 3000 msec. In the former, the visual condition posterior had more density over smaller values (i.e., was closer to the ideal observer) than the audio or audiovisual conditions. In the 3000 msec condition, the pattern reversed; the audiovisual condition had larger posterior density over smaller variance estimates than the visual and audio conditions, respectively.

To ascertain whether it was necessary to allow for different condition level parameters, we fit a constrained model in which the bisection standard deviation and metacognitive standard deviation estimates were assumed to be equivalent across the A, V, and AV conditions. We compare the models based on the Deviance Information Criteria (DIC; (Gelman et al., 2014)) which provides a measure of model fit penalized for the complexity of the model. The deviance of the posterior parameters (i.e., θ) is computed as: *D*(θ) = −2log *L*(*y*|θ), where *L*(*y*|θ) is the likelihood of the data given the model parameters. DIC is given as: $DIC=\bar{D}\left(\theta \right)+2p_{D}$, where $\bar{D}\left(\theta \right)$ is the average of the distribution of posterior deviance and *p_D_* = 2*var*[log *L*(*y*|θ)]. DIC corrects average negative log likelihood by a term which accounts for model complexity; hence, smaller values of DIC are preferred. The DICs for each condition are displayed in Table 4. As shown, the full model which allowed separate estimates for the A, V, and AV conditions was preferred in each duration condition.

**Table 4.** DIC values for the constrained and full models fit to each duration condition.

Duration	Constrained	Full
1500	−358.49	−5927.8
3000	10520	7529.9

Table 5 shows the subject-level parameters in each condition; of key interest is the subject level correlation parameter which indicates a substantial level of consistency between the precision of the interval estimates and the precision of the metacognitive decision. That is, the average posterior estimate of the correlation between the estimates for A, V, and AV is large (> 0.5) for most subjects.

**Table 5.** Subject level model parameters for each condition.

	Interval 1			Interval 2			Metacognitive			
Subject	V	A	AV	V	A	AV	V	A	AV	ρ
1500 msec duration condition										
1	0.15 (0.14, 0.16)	0.13 (0.12, 0.14)	0.12 (0.12, 0.13)	0.13 (0.12, 0.14)	0.13 (0.12, 0.14)	0.10 (0.09, 0.10)	0.40 (0.30, 0.62)	0.59 (0.40, 1.06)	0.42 (0.28, 0.83)	0.59 (0.04, 0.98)
2	0.13 (0.13, 0.14)	0.19 (0.18, 0.20)	0.10 (0.09, 0.11)	0.14 (0.13, 0.15)	0.22 (0.20, 0.23)	0.11 (0.10, 0.12)	0.62 (0.43, 1.12)	0.77 (0.50, 1.54)	0.75 (0.46, 1.70)	0.51 (0.03, 0.97)
3	0.29 (0.27, 0.32)	0.26 (0.25, 0.28)	0.17 (0.16, 0.19)	0.19 (0.18, 0.21)	0.25 (0.23, 0.26)	0.17 (0.16, 0.18)	0.30 (0.25, 0.39)	0.73 (0.51, 1.25)	0.86 (0.59, 1.59)	0.56 (0.06, 0.97)
4	0.21 (0.19, 0.22)	0.15 (0.14, 0.16)	0.09 (0.08, 0.09)	0.22 (0.21, 0.24)	0.16 (0.15, 0.17)	0.09 (0.08, 0.09)	0.83 (0.57, 1.49)	1.08 (0.70, 2.20)	0.79 (0.47, 1.95)	0.47 (0.03, 0.94)
5	0.12 (0.11, 0.13)	0.12 (0.11, 0.13)	0.08 (0.08, 0.09)	0.12 (0.11, 0.13)	0.11 (0.10, 0.12)	0.08 (0.08, 0.09)	0.41 (0.30, 0.65)	0.41 (0.28, 0.78)	0.48 (0.30, 1.01)	0.69 (0.11, 0.99)
3000 msec duration condition										
1	0.26 (0.25, 0.28)	0.20 (0.19, 0.21)	0.24 (0.23, 0.26)	0.24 (0.22, 0.25)	0.21 (0.19, 0.22)	0.21 (0.20, 0.22)	0.83 (0.60, 1.38)	1.29 (0.86, 2.52)	0.86 (0.62, 1.47)	0.62 (0.05, 0.99)
2	0.25 (0.24, 0.27)	0.21 (0.20, 0.22)	0.15 (0.14, 0.16)	0.24 (0.22, 0.25)	0.19 (0.18, 0.20)	0.16 (0.15, 0.17)	0.80 (0.56, 1.36)	1.22 (0.79, 2.47)	0.87 (0.57, 1.73)	0.60 (0.04, 0.99)
3	0.40 (0.38, 0.43)	0.40 (0.38, 0.43)	0.37 (0.35, 0.39)	0.36 (0.34, 0.38)	0.38 (0.36, 0.40)	0.31 (0.29, 0.33)	1.35 (0.96, 2.21)	1.42 (1.00, 2.46)	1.27 (0.91, 2.09)	0.83 (0.42, 1.00)
4	0.24 (0.23, 0.26)	0.18 (0.17, 0.20)	0.19 (0.18, 0.21)	0.26 (0.24, 0.27)	0.22 (0.20, 0.23)	0.19 (0.18, 0.20)	0.83 (0.59, 1.38)	1.15 (0.76, 2.28)	0.44 (0.32, 0.73)	0.50 (0.02, 0.98)
5	0.24 (0.23, 0.26)	0.14 (0.13, 0.15)	0.10 (0.10, 0.11)	0.25 (0.23, 0.26)	0.15 (0.14, 0.16)	0.12 (0.11, 0.12)	0.85 (0.60, 1.46)	0.37 (0.26, 0.73)	0.53 (0.34, 1.11)	0.33 (0.01, 0.84)

**Figure 12.**
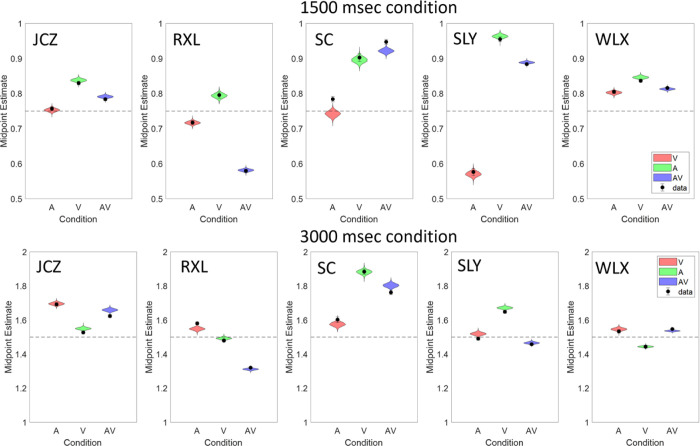
Subject-level midpoint estimates for each condition and interval duration.

To link the model estimates back into the empirical data more clearly, Figure 12 plots the posterior subject-level midpoint estimates for each condition as given by the model (the data are shown in each plot as black dots with ±1 standard error bar). These estimates qualitatively follow the same pattern as the empirical averages but are now additionally constrained by the group level estimates as well as the data from both intervals, not just the subjective “best” interval. In most cases, this additional constraint quantitatively captures the data within the 95% highest-density interval of each bisection estimate.

**Figure 13.**
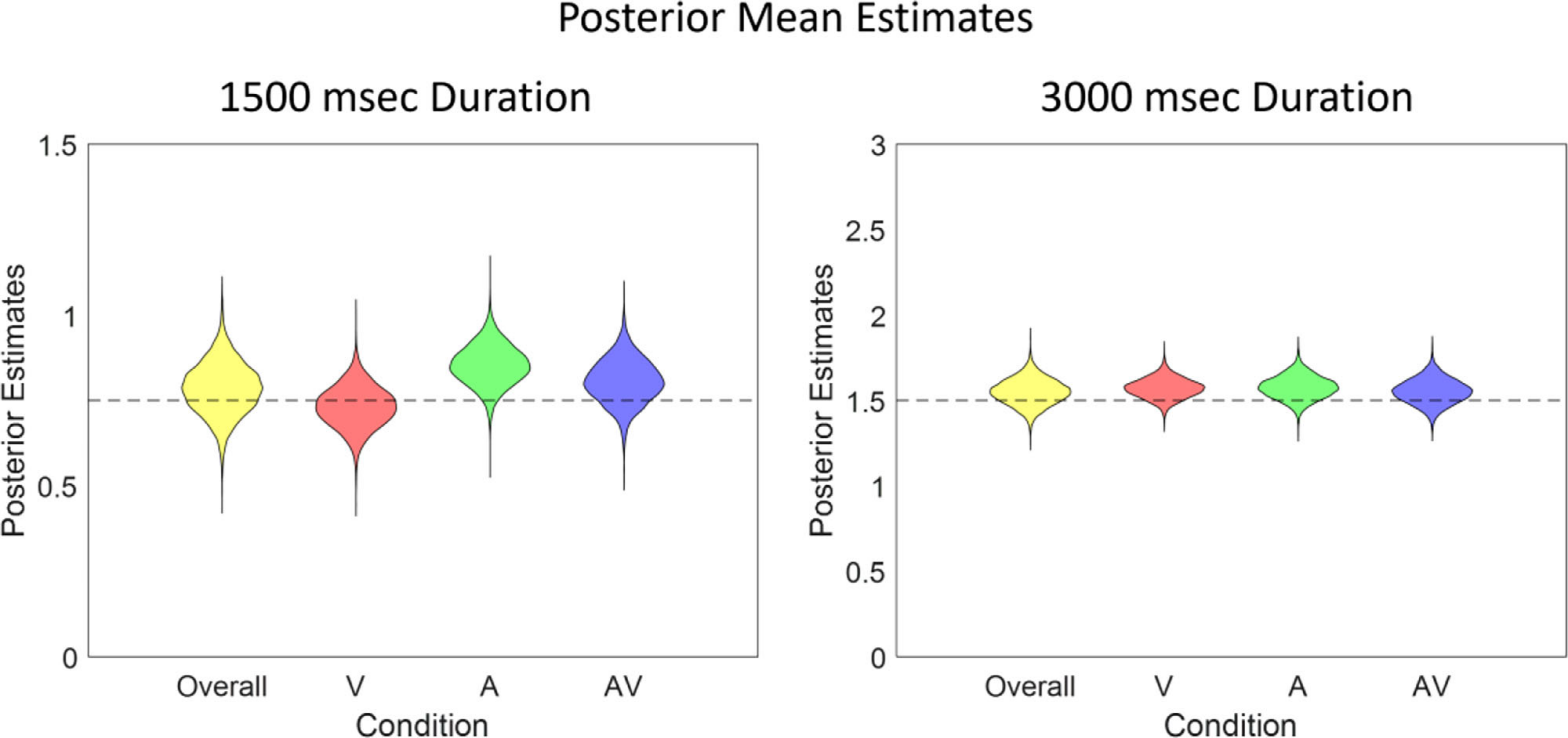
Group posterior midpoint estimates in the two interval conditions for each stimulus type and the overall mean.

The group-level midpoint estimates are shown in Figure 13; although the individual bisection estimates show noticeable variability between subjects, the group estimates tend toward the veridical bisection point. In both duration conditions, the overall mean (across modalities) tends slightly toward overestimation of the bisection interval. However, this is more consistent between modalities in the longer duration condition. In the shorter duration condition, the visual modality condition tends toward underestimation and there is greater variability between conditions, much like the empirical data.

**Figure 14.**
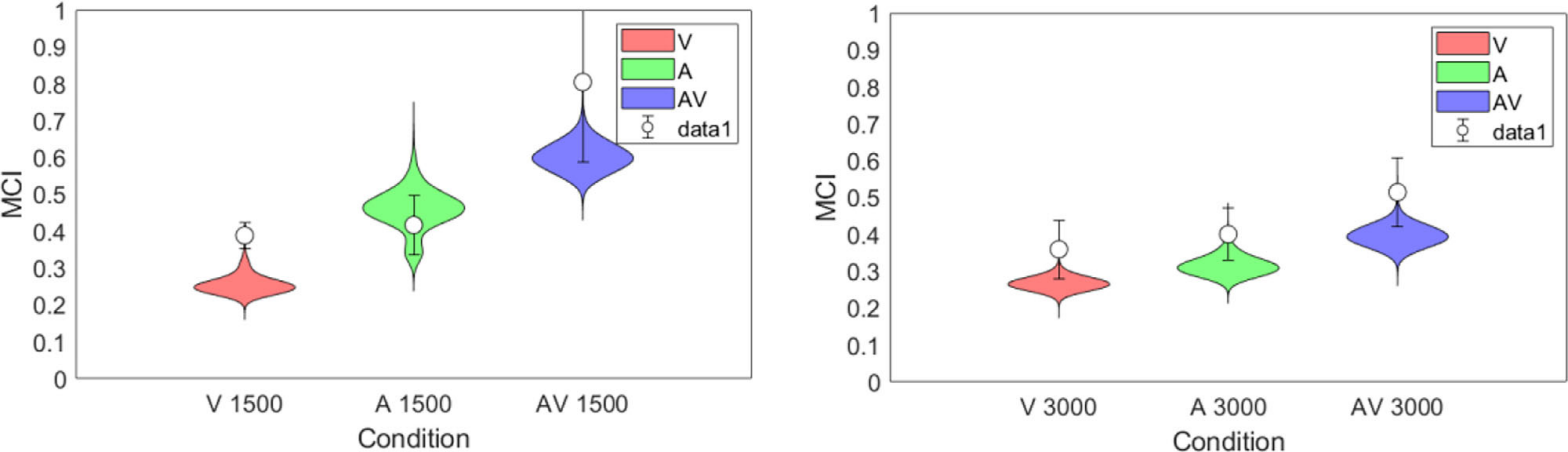
Group MCI estimated by the model shown as violin plots, with the mean group results plotted as data points. Error bars on the data points are ±1 SEM.

Finally, we compared the posterior predictive model estimates of the group MCI values with the mean values from the data in Figure 7; both results are plotted in Figure 14. That is, from the model's posterior predictions for the interval estimates and the metacognitive judgment, we derived the posterior predictions for the MCI values in the same manner as computed for the data.

The figure shows both the model and the participants have a better insight into their own performance in the short duration condition than the longer one for audio and audiovisual conditions (i.e., the posterior distribution has higher values in the A and AV conditions of the 1500 msec condition than the 3000 msec condition; 95% HDI, 1500 msec A condition = [0.33, 0.57] vs 3000 msec A condition = [0.27, 0.57]; 1500 msec AV condition = [0.53, 0.69] vs. 3000 msec AV condition = [0.34, 0.45]), the visual condition is largely similar across durations (95% HDIs, 1500 msec V condition = [0.22, 0.32] vs. 3000 msec V condition = [0.24, 0.30]). The audiovisual stimulus gives the best insight to bisection performance overall at both durations, though performance is better at the shorter duration. Although these can only be rough estimates with only five participants, it does show good agreement between the model and the empirical data, suggesting that the underlying theory of the model is a good estimate of what the participants are doing when reflecting on their performance in the interval judgement.

## Discussion

The principal aim of this article is to describe a modified paradigm to measure the perception and metacognition of time, specifically duration, and suggest why we believe it is a useful contribution to the area. We also present a model to explain the collected data over a modest range of stimulus properties and intervals. The paradigm we describe combines temporal interval *bisection* and *(re)production* as described in the existing literature (Grondin, 2010) with an additional forced-choice measure of confidence (Mamassian, 2016). In our paradigm, we ask participants to bisect (i.e., identify the mid-point of) a temporal interval as they perceive and monitor that interval, repeat the procedure, and then judge how well they performed in that pair of test intervals. This requires the subject to build up and maintain an internal representation of the target interval that they attempt to match each time. For the second-order judgement of trial-by-trial performance, where a single trial is a pair of test-intervals, it is also possible to introduce feedback to indicate whether the interval they chose to be their best estimate was, in fact, closest to the veridical midpoint of the pair. This feedback (if used) does not give any information regarding the accuracy of the estimate other than whether their choice was the better guess; there is no indication of whether that best guess was an over- or under-estimate of the veridical bisection point. This direct (first-order), ongoing behavioral estimate of duration followed by the second-order assessment of performance offers several useful and transparent ways of analyzing the collected data.

The data and modeling presented here suggest that, when the overall pattern of results is considered, although the measurement of the bisection point using this paradigm has fundamentally similar properties at both sub- and supra-second durations and across single and dual modality conditions, the metacognitive measurement is affected by both modality and duration.

There is a small underestimate of the veridical bisection point for the visual stimulus at the shorter duration compared to an overestimation for the auditory stimulus, a difference which disappears at the longer duration (Figure 6). On the face of it, this lack of a strong consistent effect of modality or duration on bisection performance is counter to reports of a shortening of perceived duration for visual stimuli compared to auditory stimuli (Droit-Volet et al., 2007; Goldstone, Boardman, & Lhamon, 1959; Goldstone & Goldfarb, 1964; Grondin, Meilleur-Wells, Ouellette, & Macar, 1998; Ortega, Lopez, & Church, 2009; Penney, Gibbon, & Meck, 2000; Walker & Scott, 1981; Wearden, Edwards, Fakhri, & Percival, 1998; Wearden, Todd, & Jones, 2006). However, our result *is* consistent with other observations, often by the same authors, showing the effect to be dependent upon many factors both stimulus and paradigm-based (Mitani & Kashino, 2016; Ortega et al., 2009; Penney et al., 2000; Wearden et al., 2006). The paradigm used here would seem to be less susceptible to audiovisual modality-based perceived duration differences than some others. It is important to note, however, we are measuring an *indirect* comparison between modalities here, although we might still have expected some systematic difference between the audio and visual midpoint estimates. This observation, however, may also give some clue to what the subjects are doing in the (admittedly unusual) task. Any shortening of perceived duration would affect the entire interval, both pre- and post-response, equally, other factors notwithstanding. If the subjects were trying to center their response in the interval, then any change in apparent duration as a product of stimulus modality would be moot; the mid-point is still the mid-point.

The focus of audiovisual interaction in time perception has generally been on the relative dominance of asynchronous signals, usually, but not always, in the favor of the auditory input (Asaoka & Gyoba, 2016; Burr et al., 2009; Chen & Yeh, 2009; de Haas, Cecere, Cullen, Driver, & Romei, 2013; Goldstone et al., 1959; Goldstone & Lhamon, 1972; Hartcher-O'Brien & Alais, 2011; Ortega et al., 2014; Ortega et al., 2009; Romei, De Haas, Mok, & Driver, 2011; van Wassenhove et al., 2008; Vicario, Rappo, Pepi, & Oliveri, 2009; Yuasa & Yotsumoto, 2015). When signals are synchronous, as in our stimuli, it has been argued that both audition and vision contribute to the duration percept in a way to optimize the precision of the result (Grassi & Pavan, 2012; Hartcher-O'Brien, Di Luca, & Ernst, 2014), and code the percept as efficiently as possible (Eagleman & Pariyadath, 2009). This also extends to some degree to a combination of audio and tactile signals (Ball et al., 2017). Our results, particularly when modeled as a group, tend to agree with this combination of both auditory and visual signals to achieve the optimal result (Hartcher-O'Brien et al., 2014), where the “AV” conditions in Figure 13 show estimates closest to the veridical midpoint of the three conditions (most obvious at 1500 msec) and a smaller standard deviation of the posterior estimates at both durations in Figure 11, indicating improved precision in the audiovisual estimate. Cautious though this suggestion must be, it is clearly not the case that combining the two signals has any detrimental effect on the duration judgement, as may have been expected were attentional resources limited.

In terms of the metacognitive response, the best insight into bisection performance is seen for the audiovisual stimulus at short durations, both for the group data and the model (Figure 14). Increasing the stimulus duration does not improve the metacognitive performance and makes it worse for audio and audiovisual modalities (visual alone stays approximately constant), though the bimodal signal still gives the best insight of the three conditions overall. That the model estimates and averaged data show such good agreement suggests the hierarchical architecture of the model bears some relationship to the underlying processes being modeled. The reduction in insight into performance with increased duration may be explained by a combination of our particular paradigm and the description of assured metacognition and confidence as internal consistency as opposed to external veridicality (Caziot & Mamassian, 2021). It has been observed that self-produced temporal intervals are perceived as being shorter and more variable than passively observed equivalents (Mitani & Kashino, 2016). In the context of a consistent stimulus regime this increased variability is confined to the suprasecond range, which would have the effect of reducing internal consistency (increasing variability) in our longer duration (self-produced) bisection task. This effect is seen in the shift to the right of the metacognitive error (σ) density curves in Figure 11 (lower) as duration increases. In the framework outlined by Caziot & Mamassian (2021), this would, in turn, result in reduced confidence and poorer metacognition of bisection performance across all stimulus modalities with longer durations, consistent with both the modeling and empirical data in Figure 14.

### Metacognition, confidence, and ideal-observer analysis

Incorporating the second-order measure of performance into the analysis allows the data to be reordered in a way that affords access to how good the subject thinks they are doing in the task. We argue that taking this objective (forced-choice) measure of how well the participant thinks they are doing in the task (i.e., how accurate they think they are) allows a more reliable measure of subjective confidence than the standard rating approach. This property is a unique aspect of the paradigm and the way in which the bisection estimate is made.

The ability to then reorder the individual trial data into perceived and actual best/worst performance allows a given subject's performance on the second-order task to be expressed in the context of an ideal observer analysis (Geisler, 1989; Geisler, 2003; Geisler, 2011) and for this relationship to be broken down over the duration of the experiment. The benefit of this paradigm for this theoretical approach is that the trial data for the ideal “device” is actual data from the subject and condition in question, rather than estimated or averaged data, as is usually the case. This means that the degree of ideal behavior of the system at the level of temporal metacognition and its properties over the duration of the experiment can be more accurately judged for a given set of conditions. It is also possible that this aspect of the data might allow some reasonable discrimination between underlying models of time perception by examining how they behave over multiple consecutive samples of a given epoch and how that compares to collected data (Jazayeri & Shadlen, 2010).

The decision made by the subjects is different from a standard two-interval forced choice magnitude-discrimination task. In the standard task, two sensory quantities are directly compared with one another, and the decision made about which is greater. In our task, each sensory quantity (i.e., the two independent bisection estimates) is (are) compared to an internal reference (the veridical, or perceived, midpoint of the interval which is never actually given) and the judgement made of which one is closer to the midpoint, making it a metacognitive judgement. The subject cannot make the correct decision based on which appears longer or shorter directly as they might both be too short (in which case the longer one is correct) or too long (the shorter one is correct). The subject still must choose which of those independent bisection decisions to accept based on their judgement of the reliability of their judgements. Although this is unique to our paradigm and any conclusions must be made in the context of the paradigm, we argue that this decision process allows us to say that we have objective evidence on accessing the information path that allows the subject to make their decision in our paradigm and we explicitly model this process.

Looking at the variance of the decisions made in this way also gives us an objective measure of observer confidence in the task and the possibility of measuring how confidence in ongoing performance changes over the duration of the experiment. This may also be considered as a way of examining internal consistency, which is possibly a better way of looking at the idea of subjective confidence in a given judgement (Caziot & Mamassian, 2021).

The addition of feedback at this level into the paradigm (i.e., only at the second-order judgement level) allows some modulation or reflection to be introduced into the metacognitive component (or in Bayesian terms, a prior to be updated). The only noticeable effect of this adding feedback on subjects’ bisection performance was to decrease the number of trials at which a bisection judgement somewhat close to veridical was reached (25-50 trial pairs in most subjects (Corcoran et al., 2018)). An unusual property of providing feedback at this stage in the decision process is that once the perceived bisection point is estimated relatively accurately in both intervals, they potentially become indiscriminable from one another making the provision of feedback more influential on the decision process earlier in the trial series.

### Modeling

The benefit of the hierarchical Bayesian model is that it allows for simultaneous estimation of group- and observer-level parameters and provides an estimate of the uncertainty in those parameters. Hierarchical Bayesian analysis is a method used to good effect in complex multilevel systems such as ocean ecology where each level of analysis informs and influences the others in a continuous manner and each dataset has its own particular set of conditions (Britten et al., 2021). Given what we are trying to measure here, although not as complex as oceanic carbon fluctuations across the globe, we consider the first- and second-order decisions inherent in our method to fit the hierarchical approach.

In our implementation, we jointly estimate the variability of the interval estimation and the metacognitive estimates allowing for determination of the correlation in the error between these estimates. Our model is a measurement model rather than a psychological model, using the data to return useful estimates of uncertainty parameters. As such it is an effective form of statistical analysis of the data rather than a standard descriptive model more common in the literature. Nevertheless, we model the decision process in a psychologically-meaningful way (see Figure 10) drawing on principles of signal detection (Green & Swets, 1966; Macmillan & Creelman, 1991) to provide a theoretical framework for the decision. This approach provides a good fit between the data and the model estimates for both the bisection and the metacognitive task, which encourages us in our approach. We have deliberately kept it as simple as possible, but we consider this a strength in our implementation. A limitation of our approach, however, is that we assume the psychological midpoint is static across trials (contra to Figure 3). The model could therefore be extended by allowing the estimates to vary functionally across trials, as measured for each participant, but we leave that for future work.

An interesting feature of these data and the model is that although there is substantial individual difference in performance, the performance of the group when modeled in this way approaches the external veridical bisection point, suggesting a shared sense of time despite the obvious subjective differences; a time-based version of Aristotle's “wisdom of the crowd” (Galton, 1907), and a possible representation of our shared thread of “real” time.

## Conclusions

The paradigm described here offers a different way to measure the percept of brief intervals of time as they are experienced by the subject. The two stages of the decision give both a first-order measure of the temporal percept and a second-order metacognitive judgement of performance. The summary measures provide some equivalence to the extensive literature on time perception, while the more unique trial-by-trial data collection affords a detailed window into the way in which the system builds up a representation of the duration to be bisected, which in turn mediates future performance.

Time and our insight into its passing has stimulated debate for centuries from many disparate perspectives and continues to do so, an observation illustrated nicely in recent theoretical articles and the associated commentaries (Buonomano & Rovelli, 2022; Glicksohn, 2022; Grondin, 2023; Gruber et al., 2022; Miller & Wang, 2022). We consider that measuring both the cognition and metacognition of sense of time passed in the way illustrated here will contribute positively to that debate.

## Footnotes

1 As pointed out by one of the reviewers, this is not unique to time because both number and space may be derived from several sensory inputs. We do, however, consider time to be a particularly primal sense to be unbound by modality and interesting because of this.

2 Bayes factors (BF_10_) may be interpreted as follows: 1, No effect; 1–3, Anecdotal/Not worth more than a bare mention; 3–10, Moderate; 10–30, Strong; 30–100, Very Strong; >100 Extreme. (After Jeffreys, 1961; Kass & Raftery, 1995; Lee & Wagenmakers, 2014).

## Appendix A: Appendix A: Additional data for the Bayesian repeated-measures ANOVAs

For the ANOVAS, all models include subject, and random slopes for all repeated measures factors. For the post-hoc comparisons, the posterior odds have been corrected for multiple testing by fixing to 0.5 the prior probability that the null hypothesis holds across all comparisons (Westfall, Johnson, & Utts, 1997). Individual comparisons are based on the default *t* test with a Cauchy (0, r = 1/sqrt(2)) prior. The “U” in the Bayes factor denotes that it is uncorrected (JASP, Version 0.18).

### Subjective “best” bisection estimates

#### Individual planned comparisons for each duration

**Table:**

Models	P(M)	P(M|data)	BF_M_	BF_10_	Error %
Model comparison 1500 msec					
Null model (incl. subject and random slopes)	0.500	0.086	0.094	1.000	
Condition	0.500	0.914	**10.686**	10.686	0.602
Model comparison 3000 msec					
Null model (incl. subject and random slopes)	0.500	0.738	2.813	1.000	
Condition	0.500	0.262	0.356	0.356	0.739

#### Post-hoc tests for the bisection ANOVA

Combined ANOVA for the “best” bisection estimate (Figure 6 in main text):

**Table:**

Post Hoc Comparisons	Prior Odds	Posterior Odds	BF_10, U_	Error %
Condition				
Visual				
Audio	0.587	0.390	0.664	0.016
Audiovisual	0.587	0.486	0.828	0.019
Audio				
Audiovisual	0.587	0.266	0.453	0.010
Duration				
1500–3000 msec	1.000	0.301	0.301	0.012

Individual ANOVA for each duration - bisection:

**Table:**

Post Hoc Comparisons	Prior Odds	Posterior Odds	BF_10, U_	Error %
Condition 1500 msec				
Visual				
Audio	0.587	0.730	1.242	0.019
Audiovisual	0.587	1.278	2.175	3.850 × 10^−4^
Audio				
Audiovisual	0.587	1.844	3.140	4.188 × 10^−4^
Condition 3000 msec				
Visual				
Audio	0.587	0.235	0.400	0.001
Audiovisual	0.587	0.277	0.472	0.004
Audio				
Audiovisual	0.587	0.275	0.468	0.004

### Metacognitive Index (MCI) Estimates

#### Individual planned comparisons for each duration

**Table:**

Models	P(M)	P(M|data)	BF_M_	BF_10_	Error %
Model comparison 1500 msec					
Null model (incl. subject and random slopes)	0.500	0.117	0.132	1.000	
Condition	0.500	0.883	7.568	7.568	0.586
Model comparison 3000 msec					
Null model (incl. subject and random slopes)	0.500	0.502	1.007	1.000	
Condition	0.500	0.498	0.993	0.993	0.595

#### Post hoc tests for the MCI ANOVA

Combined ANOVA for the MCI estimate (Figure 7 in main text):

**Table:**

Post Hoc Comparisons	Prior Odds	Posterior Odds	BF_10, U_	error %
Condition				
Visual				
Audio	0.587	0.587	0.999	0.022
Audiovisual	0.587	6.334	10.783	1.044 × 10^−5^
Audio				
Audiovisual	0.587	0.726	1.236	0.026
Duration				
1500–3000 msec	1.000	0.484	0.484	0.017

Individual ANOVA for each duration - MCI:

**Table:**

Post Hoc Comparisons—Condition	Prior Odds	Posterior Odds	BF_10, U_	error %
1500 msec				
Visual				
Audio	0.587	86.470	147.208	1.382 × 10^−4^
Audiovisual	0.587	2.162	3.681	1.491 × 10^−4^
Audio				
Audiovisual	0.587	0.415	0.707	0.011
3000 msec				
Visual				
Audio	0.587	0.256	0.435	0.002
Audiovisual	0.587	0.649	1.105	0.016
Audio				
Audiovisual	0.587	0.444	0.756	0.012

## Appendix B: JAGS code for the Hierarchical Bayesian model

model{

 # Variance of precision draws

 hyperPrec ∼ dunif(0, 100)

 hyperSigma <- 1/sqrt(hyperPrec)

 # Midpoint estimates

 mpprec ∼ dunif(0, 100)

 groupMidpoint ∼ dnorm(midpoint, mpprec)

 # Cycle over conditions

 for (k in 1:ncons){

  conprec[k] ∼ dunif(0,100) # Condition precision

  conmidpoint[k] ∼ dnorm(groupMidpoint, conprec[k]) # midpoint estimate for each condition

  ## Group level precision means

  # interval 1 estimate

  mprec1[k] ∼ dunif(0, 100)

  logmprec1[k] <- log(mprec1[k]) # Log transform

  msigma1[k] <- 1/sqrt(mprec1[k]) # convert to standard deviation

  # interval 2 estimate

  mprec2[k] ∼ dunif(0, 100)

  logmprec2[k] <- log(mprec2[k])

  msigma2[k] <- 1/sqrt(mprec2[k])

  # metacognitive estimate

  metaLambda[k] ∼ dunif(0, 100)

  logMetaLambda[k] <- log(metaLambda[k])

  metasigma[k] <- 1/sqrt(metaLambda[k])}

 }

 # Cycle over subjects

 for (j in 1:nsubjects){

  # subject specific correlation between conditions

  subr[j] ∼ dunif(0, 1)

  # Build covariance matrix from standard deviation

  Sigma[1,1,j] <- pow(hyperSigma,2) + .1

  Sigma[1,2,j] <- subr[j] * pow(hyperSigma,2)

  Sigma[1,3,j] <- subr[j] * pow(hyperSigma,2)

  Sigma[2,1,j] <- subr[j] * pow(hyperSigma,2)

  Sigma[2,2,j] <- pow(hyperSigma,2) + .1

  Sigma[2,3,j] <- subr[j] * pow(hyperSigma,2)

  Sigma[3,1,j] <- subr[j] * pow(hyperSigma,2)

  Sigma[3,2,j] <- subr[j] * pow(hyperSigma,2)

  Sigma[3,3,j] <- pow(hyperSigma,2) + .1

  # convert to precision matrix

  Omega[1:ncons,1:ncons,j] <- inverse(Sigma[ , ,j]) # invert matrix for dmnorm

  # sample log precision estimates for A, V, and AV for

  logsubmprec1[j,1:ncons] ∼ dmnorm(logmprec1[1:3], Omega[ , ,j]) # interval 1

  logsubmprec2[j,1:ncons] ∼ dmnorm(logmprec2[1:3], Omega[ , ,j]) # interval 2

  logSubMetaLambda[j,1:ncons] ∼ dmnorm(logMetaLambda[1:3], Omega[ , ,j]) # metacognitive decision

  # possible problem is that precision samples can be negative so transform them back to I[0, ]

  # cycle over conditions

  for (m in 1:ncons){

   subprec[j,m]∼ dunif(0, 100)

   submid[j,m] ∼ dnorm(conmidpoint[m], subprec[j,m]) # subjective midpoint esetimates

   # subject precisions for

   submprec1[j,m] <- exp(logsubmprec1[j,m]) # interval 1

   submprec2[j,m] <- exp(logsubmprec2[j,m]) # interval 2

   subMetaLambda[j,m] <- exp(logSubMetaLambda[j,m]) # metacognitive decision

  }

 }

 # Cycle through each trial

 for (i in 1:ndata){

  # Likelihood of bisection estimate

  # mprec indicates the variability of the midpoint estimate

  # (smaller indicates a more accurate midpoint estimate)

  i1[i] ∼ dnorm(submid[s[i], c[i]], submprec1[s[i], c[i]])

  i2[i] ∼ dnorm(submid[s[i], c[i]], submprec2[s[i], c[i]])

  # Subjective error

  d1[i] <- abs(i1[i] - submid[s[i], c[i]])

  d2[i] <- abs(i2[i] - submid[s[i], c[i]])

  # Estimated error difference

  dhat[i] <- d1[i] - d2[i]

  # Find probability of responding with interval 1

  # Lambda indicates the variability of the error estimate

  # Lower values indicate a more accurate assessment of error

  p[i] <- pnorm(0, dhat[i], subMetaLambda[s[i], c[i]]) # Integrate the difference distribution up to 0

  # Likelihood of metacognitive choice

  r[i] ∼ dbern(p[i]) # Each metacognitive choice is a Bernoulli variable with a probability of selecting interval 1 specified by p

 }

}
